# Hollow Li_20_B_60_ Cage: Stability and Hydrogen Storage

**DOI:** 10.1038/srep24500

**Published:** 2016-04-14

**Authors:** Jing Wang, Zhi-Jing Wei, Hui-Yan Zhao, Ying Liu

**Affiliations:** 1 Department of Physics and Hebei Advanced Thin Film Laboratory, Hebei Normal University, Shijiazhuang 050024, China; 2 State Key Laboratory for Superlattices and Microstructures, Institute of Semiconductors, Chinese Academy of Sciences, Beijing 100083, China; 3 National Key Laboratory for Materials Simulation and Design, Beijing 100083, China

## Abstract

A stable hollow Li_20_B_60_ cage with *D*_2_ symmetry has been identified using first-principles density functional theory studies. The results of vibrational frequency analysis and molecular dynamics simulations demonstrate that this Li_20_B_60_ cage is exceptionally stable. The feasibility of functionalizing Li_20_B_60_ cage for hydrogen storage was explored theoretically. Our calculated results show that the Li_20_B_60_ molecule can adsorb a maximum of 28 hydrogen molecules. With a hydrogen uptake of 8.190 wt% and an average binding energy of 0.336 eV/H_2_, Li_20_B_60_ is a remarkable high-capacity storage medium.

Boron is an element of fascinating chemical complexity due to the multicenter bonds, which yield a wide range of boron structures from the usual three dimensional (3D) polyhedral geometries^1^ to one-dimensional (1D) nanotubes^2^,^3^ and the recently discovered B_80_ cage^4^, which is structurally analogous to C_60_ with 12 pentagonal and 20 hexagonal rings. The B_80_ cage has an additional boron atom at the center of each hexagon, and has the same icosahedral point group symmetry as does the C_60_ fullerene. Based on first-principles calculations, several other fullerenes and stuffed fullerene-like structures have been proposed. Yan *et al.*^5^ constructed a series of stable B_80+8*k*_ (0 ≤ *k* ≤ 5, *k* ≠ 1) fullerenes by using the modified leapfrog algorithm. Zope *et al.*^6^ demonstrated the existence of a family of stable boron fullerenes containing 80*n*^2^ (*n* = 1–5) atoms. Prasad *et al.*^7^ found that for B_98_, B_99_, B_100_, B_101_, and B_102_ clusters, the stuffed fullerenes built on icosahedral-B_12_ units, are more stable than the fullerene-like boron clusters.

In the course of ongoing studies of the geometric structures of B_*n*_ clusters, the design of boron-based nanomaterials is being closely examined for their applications in hydrogen storage^8^,^9^,^10^,^11^,^12^. Using first-principles calculations, the buckyball B_80_ coated with various metals *M* = Li, Na, K, Be, Mg, Ca, Sc, Ti, and V has been investigated for hydrogen storage^8^,^9^,^12^. It has been found that Na and Ca appear to be the best candidates for hydrogen storage. The B_80_Na_12_ fullerene can store up to 72 H_2_ molecules with a gravimetric density of 11.2%^8^ while Ca_12_B_80_ can bind up to 66 H_2_ molecules with a hydrogen storage capacity of 9.0 wt%^9^. The hydrogen storage properties of planar boron sheets coated with alkali metals have also been investigated, and the boron-Li system was found to be a good candidate for hydrogen storage purposes^10^. *Ab initio* studies of hydrogen adsorption in Li-doped hexagorane (B_6_H_6_Li_2_) were carried out by Srinivasu *et al.*^11^. They found each Li site can adsorb a maximum of three hydrogen molecules which corresponds to a gravimetric density of 12 wt%. More recent investigations of boron nanostructures have shown that Li-decorated boron sheets and nanotubes based on the boron double ring are potential hydrogen storage media^12^. These studies indicate that there is an exciting future for boron-based nanomaterials, specifically in low dimensional structures, which might lead to novel devices with diverse and unique properties^13^.

In the present work, we report that our first-principles calculations within density functional theory (DFT) have identified a stable Li_20_B_60_ cage. We have explored the feasibility for hydrogen storage, and the results show that the hydrogen uptake of the Li_20_B_60_ molecule is 8.190 wt%, making it an attractive candidate as a high-capacity hydrogen storage material.

A large Li_20_B_60_ cage was constructed based on a Li-B sheet as shown in Fig. 1, which is stable and can maintain its original 2D configuration. After full relaxation, it was found that the Li_20_B_60_ cage obtained had robust stability and a nearly spherical shape with a D_2_ point group symmetry, as shown in Fig. 2(a). The binding energy of Li_20_B_60_ cage is −4.888 eV/atom, which is closely related to that of the Li-B sheet (−4.957 eV/atom). In this cage structure, the B atoms can be seen to form “truncated octahedrons” with the 20 Li atoms capping the fourteen faces. For the Li atoms, there are three different positions: six “face-centered” sites (Li^*I*^), four top sites on the “truncated faces” formed by the boron hexagons (Li^*II*^), and four top sites on the “truncated faces” formed by boron triangles (Li^*III*^). On each “face-centered” site, there exists a Li_2_ dimer with an average Li-Li distance of 2.635 Å. Four Li atoms are located on the top sites of the “truncated face” formed by the boron hexagons. The remaining 4 Li atoms are located on the top sites of the boron triangles. We refer to these atoms below as Li^*I*^, Li^*II*^, and Li^*III*^, respectively, as shown in Fig. 2. The relative stability of this Li_20_B_60_ cage was discussed by comparing with other structures, which arose during the high-temperature dynamic simulations, but no lower-energy structures were found (*see* Figures S2 and S3 in the Supplementary Information).

The stability of Li_20_B_60_ was further checked using vibrational frequency analysis and molecular dynamics (MD) simulations. The vibrational frequency analysis of the Li_20_B_60_ cage indicates no imaginary frequencies and the highest intensity frequency was 687.9 *cm*^−1^. For more details see Section IV of the Supplementary Information, as well as some low-frequency modes. Therefore, the Li_20_B_60_ cage is kinetically stable. We also carried out *ab initio* molecular dynamics simulations with the constant-temperature, constant-volume (NVT) ensemble in a Massive Nosè-Hoover thermostat. The total simulation time was set to be 1.0 *ps* with 1000 dynamics steps. It was found that the structure of the D_2_-Li_20_B_60_ cage was not disrupted up to a temperature of ~600 K. These results indicate that D_2_-Li_20_B_60_ cage has good thermodynamic stability.

It is natural to explore the electronic structure of the Li_20_B_60_ cage. To this end, we calculated the deformation electron density, partial density of states (PDOS), and frontier molecular orbitals, including the highest occupied molecular orbital (HOMO) and the lowest unoccupied molecular orbital (LUMO) as shown in Figs 2(*b*) and 3. From the deformation electron density, one can see an alternation of three-center and two-center bonds on each of the six centered-faces of the “truncated octahedron”. On the “truncated faces” formed by the boron hexagons, there is obvious *sp*^2^-like bonding between B atoms, while on the “truncated faces” formed by the boron triangles, the Li atoms contribute parts of their 2*s* electrons to the neighboring B atoms. From the point of view of doping, the three-center triangular regions could be regarded as donors and the two-center hexagonal regions could be regarded as acceptors^14^. Thus it is the mixing of the two-center and three-center bonding that promotes the stability of the D_2_-Li_20_B_60_ cage. As for the case of the HOMO and LUMO orbitals, it was found that the HOMO orbitals are mostly localized on the B atoms and have a predominantly *s*-*p* hybridization characteristic. The LUMO orbitals are also mostly localized on the B atoms and the hybridization is also predominantly of *s*-*p* character, which is consistent with the hybridization of Li-B sheet^15^. The energy level features show that the LUMO orbital is doubly degenerate. Close examination of the PDOS (*see* Fig. 3) further confirms the hybridization characteristics of the HOMO and LUMO orbitals.

We next investigated the interaction between the Li_20_B_60_ cage and hydrogen molecules. It was found that hydrogen can bind to the Li sites with a binding strength reflecting typical van der Waals interactions. The hydrogen binding energy (*E*_*b*_) for Li_20_B_60_ is defined as *E*_*b*_ = {*E*(*Li*_20_*B*_60_) + *n* × *E*(*H*_2_) − *E*[*Li*_20_*B*_60_(*H*_2_)_*n*_]}/*n*. We first added one H_2_ molecule near each Li atom. After energy minimization, it was found that the H_2_ molecule tends to occupy a position above the Li atom and with its axis parallel to the boron hexagonal or triangle plane. The average distance of the H_2_ molecule from the Li atom is 2.270 Å indicating a van der Waals interaction between the H_2_ molecules and the Li_20_B_60_ cage. The average adsorption energy of the first adsorbed H_2_ molecule of each Li is 0.460 eV which lies within the range 0.1–0.6 eV suggested as a criterion for a hydrogen storage medium.

A second and third H_2_ were then added. The results indicated that the Li atoms in different positions can adsorb different numbers of H_2_ molecules. Each Li^*I*^ or Li^*II*^ atom can only adsorb one H_2_, and the H_2_ prefers to be located right above the Li atom. The Li^*III*^ atom can absorb a maximum of three H_2_ molecules. Thus, a total of 28 hydrogen molecules can be adsorbed onto the surface of the Li_20_B_60_ system as shown in Fig. 4, corresponding to a hydrogen uptake of 8.190 wt% with an average binding energy of about 0.336 eV/H_2_. The binding energies of all 28 H_2_ molecules have also been counted and the values distribute in the range 0.1–0.4 eV/H_2_ (*see* Figure S1 of Supplementary Information). This hydrogen storage capacity is in excess of 6 wt%, the U. S. Department of Energy target and is comparable to some similar systems, such as the alkali-metal(Li, Na, K)-doped B_80_ fullerenes^8^, the Li-doped boron sheet^13^,^15^ and boron nanotubes^13^,^15^. All these results suggest that the Li-B cage is a potential candidate for hydrogen storage.

In summary, our first-principles studies have identified a stable Li_20_B_60_ molecule. The results of vibrational frequency analysis and molecular dynamics simulations demonstrate that this Li_20_B_60_ cage is exceptionally stable. The Li_20_B_60_ cage can adsorb a maximum of 28 H_2_ molecules, resulting in a hydrogen gravimetric density of 8.190 wt% with an average adsorption energy of 0.336 eV/H_2_. This is a remarkable result indicating another application for the Li-B cage as a potential high-capacity storage medium.

## Methods

Our calculations were carried out with the exchange-correlation potential described by the Perdew-Burke-Ernzerhof version (PBE) of the general gradient approximation (GGA)^16^, as implemented in the DMol^3^ package^17^. The double-numerical basis plus polarized functions (DNP) was chosen. When discussing the adsorption of hydrogen molecules onto the Li_20_B_60_ cage, the van der Waals (vdW) interactions^18^, which are crucial for the formation, stability, and function of molecules were taken into account. Here, the hybrid semi empirical dispersion-correction approach of Tkatchenko and Scheffler (TS) scheme^19^, was used in the process of structure optimization. Some previous studies^13^,^15^,^20^ have investigated the hydrogen capacity of metal-decorated 2D sheets or 1D nanotubes using the semi-empirical dispersion-correction approach. All structures were fully relaxed and geometric optimizations were performed with convergence thresholds of 10^−5^ hartree (Ha) for the energy, 2 × 10^−3 ^Ha/Å for forces, and 5 × 10^−3^ Å for the atomic displacements. In the self-consistent field calculations, the convergence threshold was set to 10^−6 ^Ha on the total energy. Geometry optimizations were performed with unrestricted spin and without any symmetry constraints.

## Additional Information

**How to cite this article**: Wang, J. *et al.* Hollow Li_20_B_60_ Cage: Stability and Hydrogen Storage. *Sci. Rep.* **6**, 24500; doi: 10.1038/srep24500 (2016).

## Supplementary Material

Supplementary Information

## Figures and Tables

**Figure 1:**
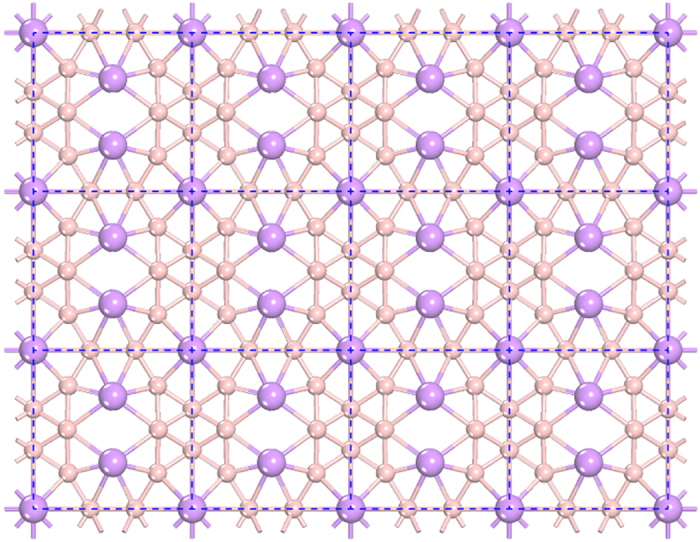
Configuration of the Li-B sheet. Dark (purple) ball: Li atom; light (prink) ball: B atom.

**Figure 2:**
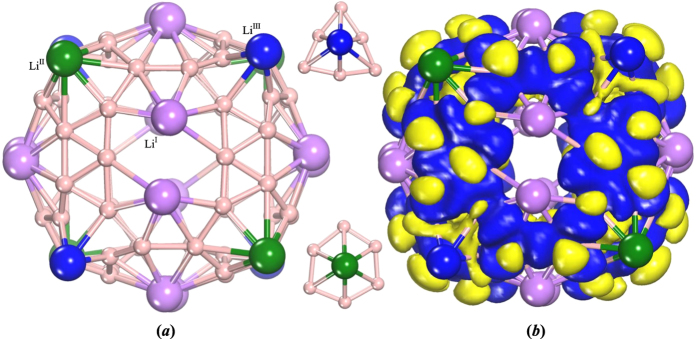
(**a**) Configuration and (**b**) deformation electron density for the Li_20_B_60_ cage. In part (**a**), the purple, green and blue balls show the Li^*I*^, Li^*II*^ and Li^*III*^ atoms, respectively and the two inside pictures explain the surrounding structures of Li^*II*^ and Li^*III*^. In part (**b**), the blue and yellow regions represent the positive and negative values of the charge density and the isosurface corresponds to 0.03 e/Å^3^.

**Figure 3:**
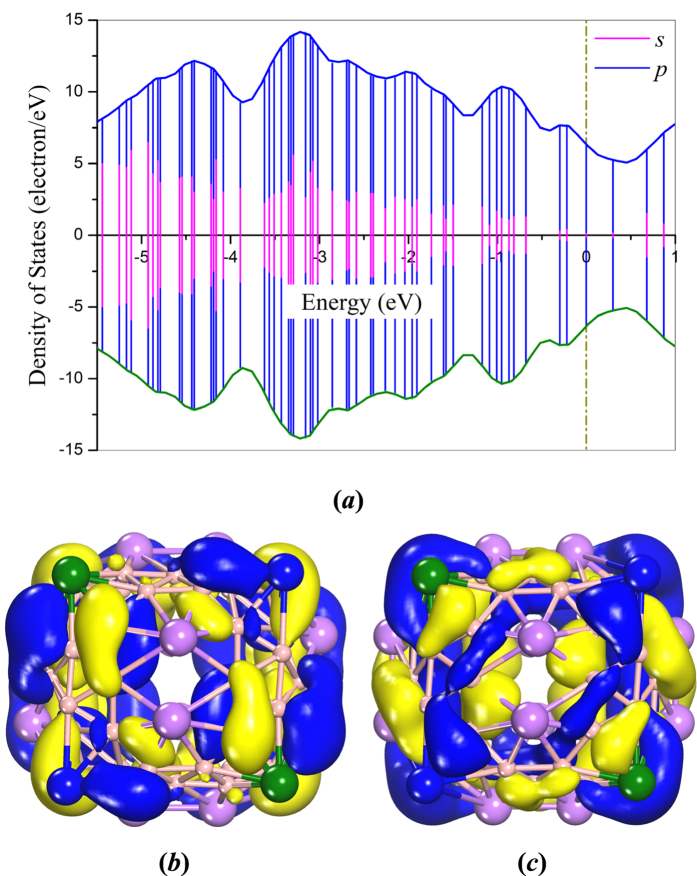
(**a**) Partial density of states (PDOS), (**b**) HOMO and (**c**) LUMO orbitals for Li_20_B_60_ cage. In part (**a**), the positive and negative DOSs represent spin up and spin down. In parts (**b**,**c**), different colors represent the phases of wave functions: blue for positive and yellow for negative. The isosurface is set at a value of 0.015 e/Å^3^.

**Figure 4:**
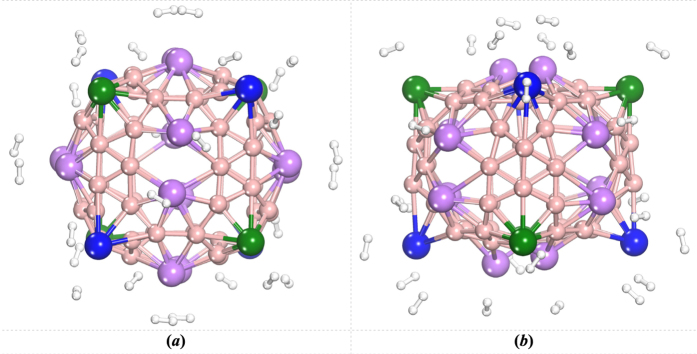
Optimized structure of Li_20_B_60_ cage with hydrogen molecules adsorbed by the Li atom. (**a**,**b**) view from different angles.
