# Supplementary material for: Hollow Li20B60 Cage: Stability and Hydrogen Storage
Source: Sci Rep. 2016 Apr 14;6:24500. doi: 10.1038/srep24500 (PMC4830930; doi:10.1038/srep24500)
Supplement: Supplementary Information [file srep24500-s1.doc]

Hollow Li_20_B_60_ Cage: Stability and Hydrogen Storage

Jing Wang^1,2^, Zhi-Jing Wei^1^, Hui-Yan Zhao^1^, and Ying Liu^1,3^,*

^1^Department of Physics and Hebei Advanced Thin Film Laboratory, Hebei Normal University, Shijiazhuang 050024, Hebei, China

^2^State Key Laboratory for Superlattices and Microstructures, Institute of Semiconductors, Chinese Academy of Sciences, Beijing 100083, China

^3^National Key Laboratory for Materials Simulation and Design, Beijing 100083, China

**Section I. Binding energies of 28 H_2_ molecules**

|  |
| --- |
| **Figure S1.** The statistical histogram of the binding energies of 28 H_2_ molecules. It visually demonstrates the distribution of binding energies and the values are all in the range 0.1-0.4 eV/H_2_. Here the binding energy is defined by *E*_b_(H_2_)= *E*[Li_20_B_60_(H_2_)_27_]+*E*(H_2_)-E[Li_20_B_60_(H_2_)_28_]. |

**Section II. Relative stability**

| 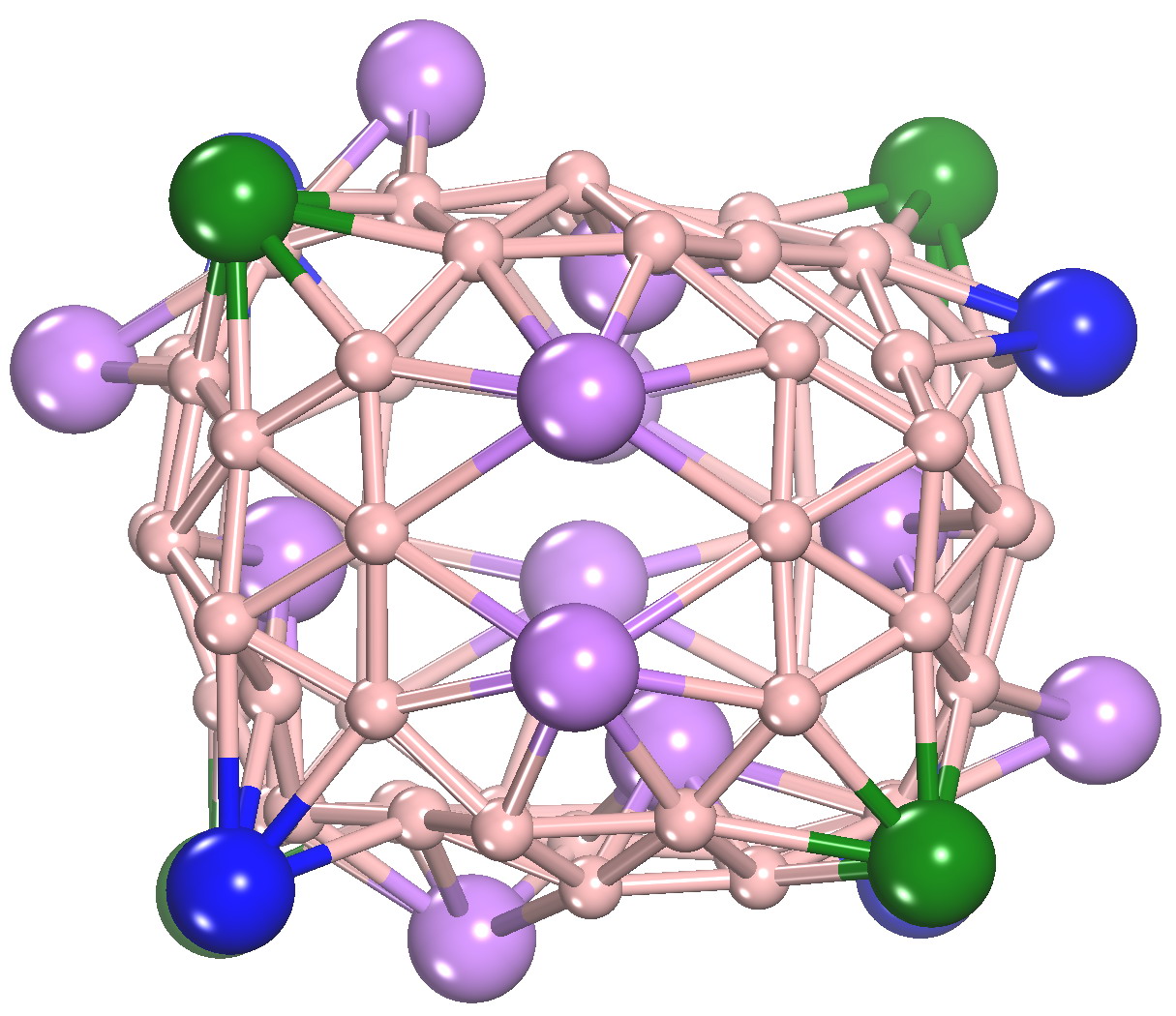 | 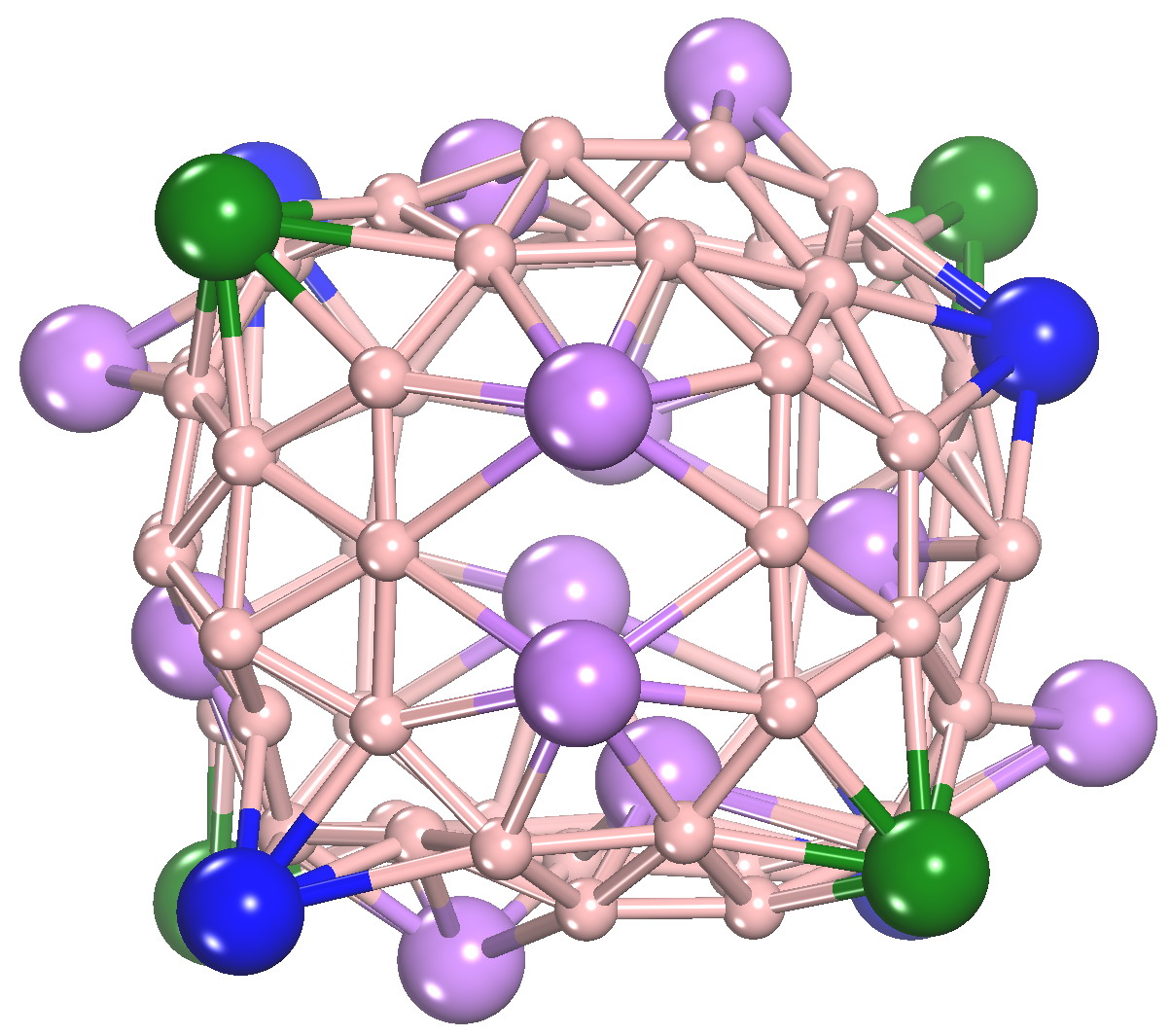 | 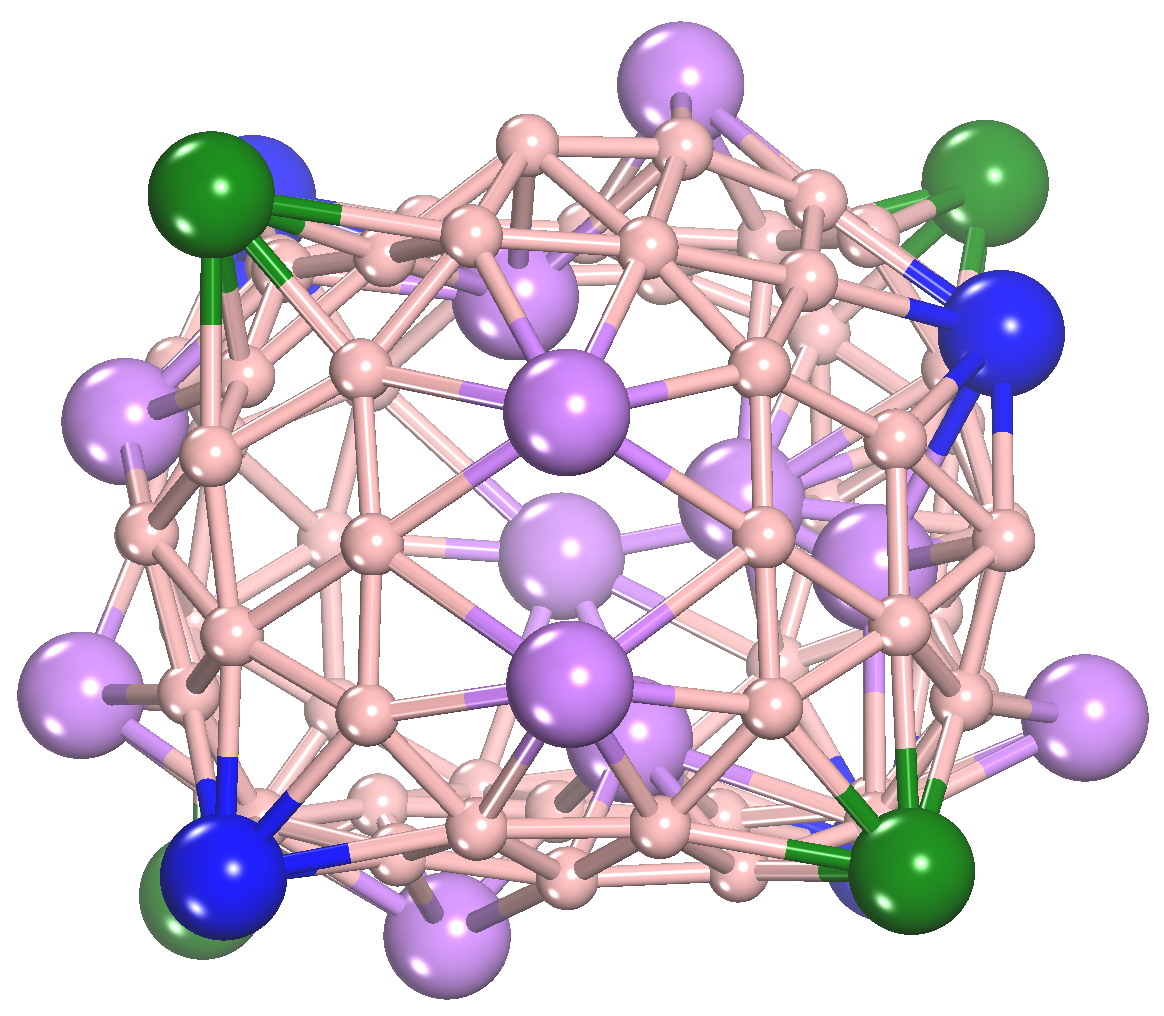 | 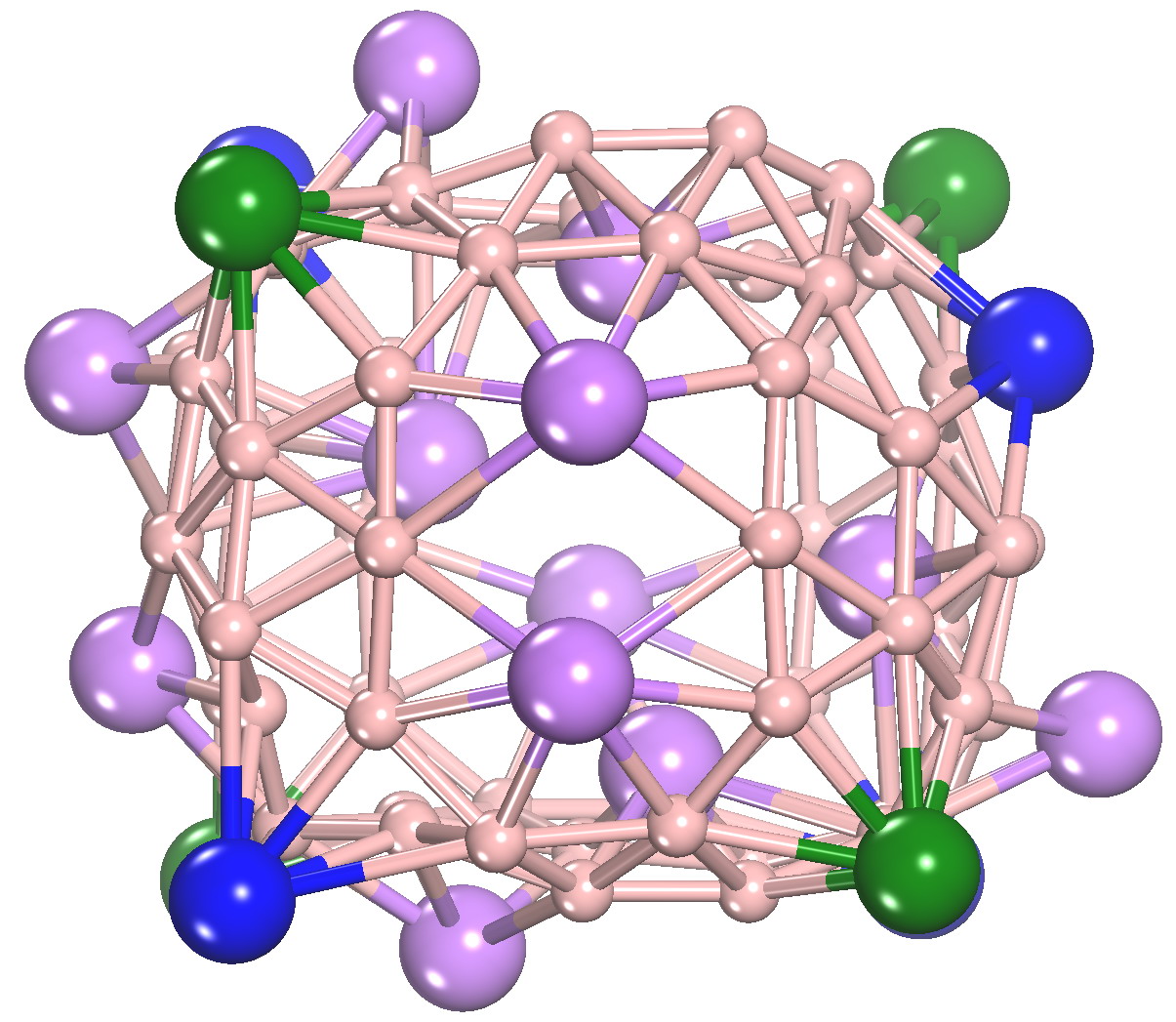 |
| --- | --- | --- | --- |
| (*a*) NVE-3000K-78*th*  ∆*E*= 2.259eV | (*b*) NVE-3000K-101*th*  ∆*E*= 1.189eV | (*c*) NVE-3000K-221*th*  ∆*E*= 1.268eV | (*d*) NVE-3000K-235*th*  ∆*E*= 1.790eV |
| 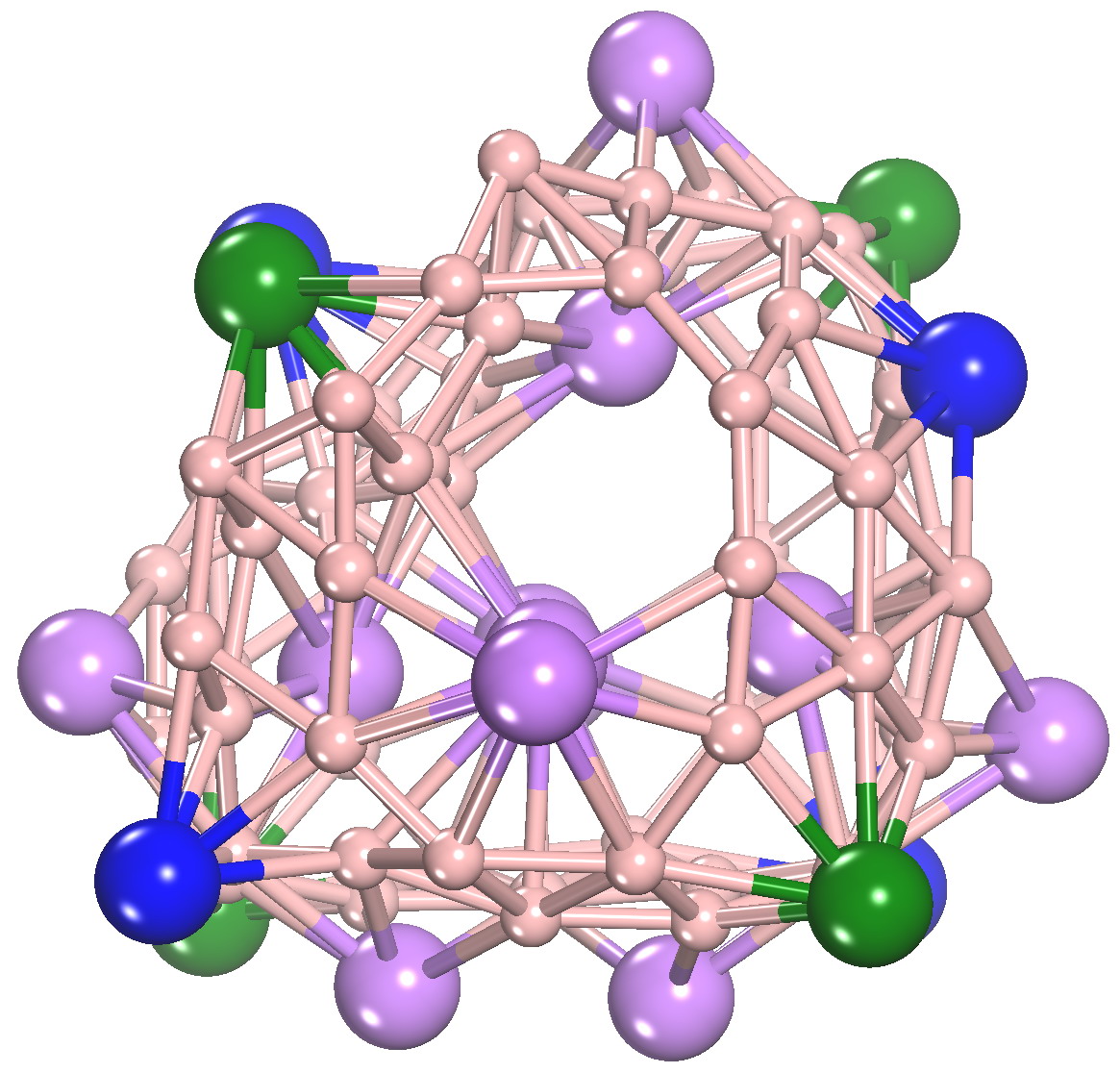 | 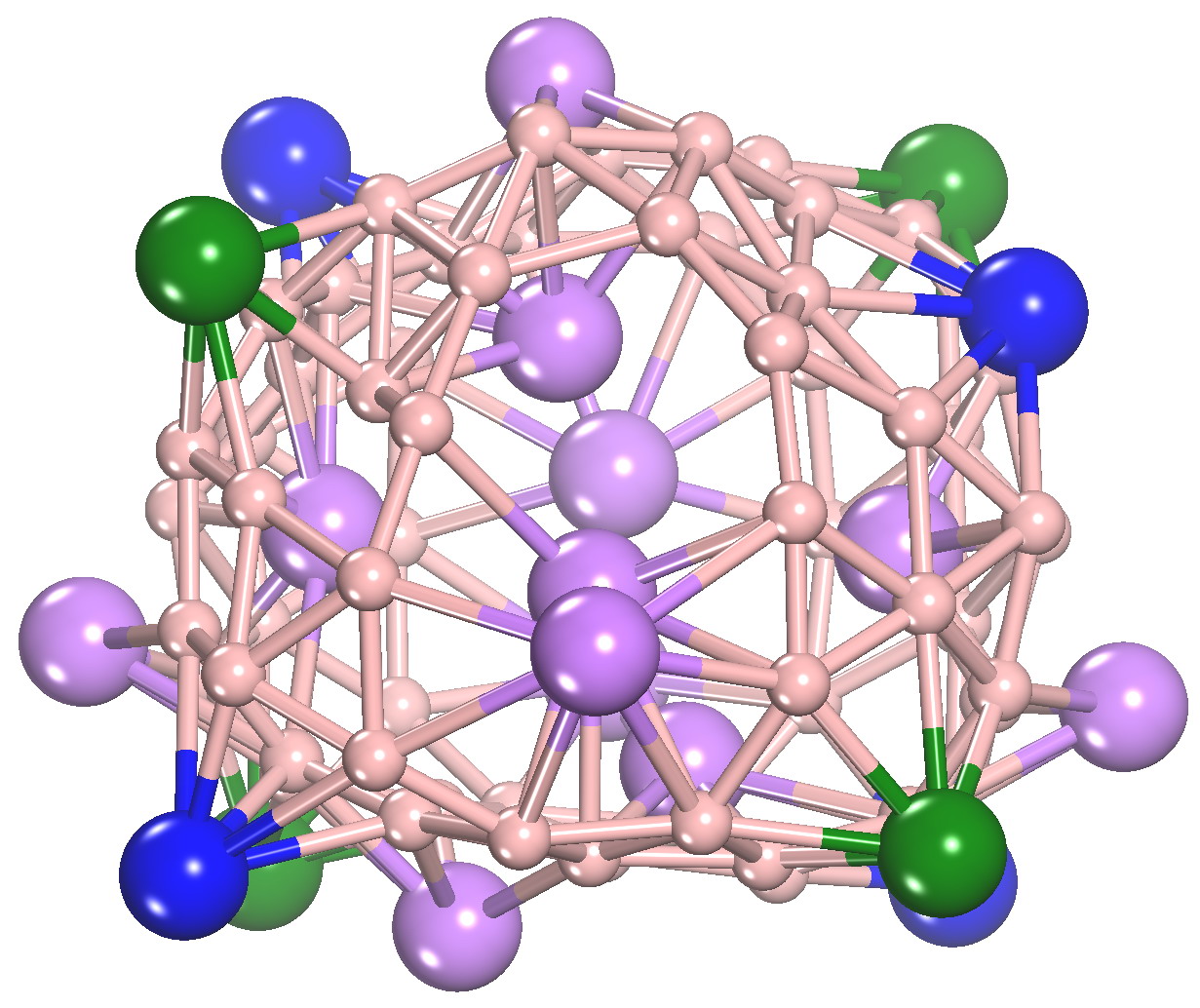 | 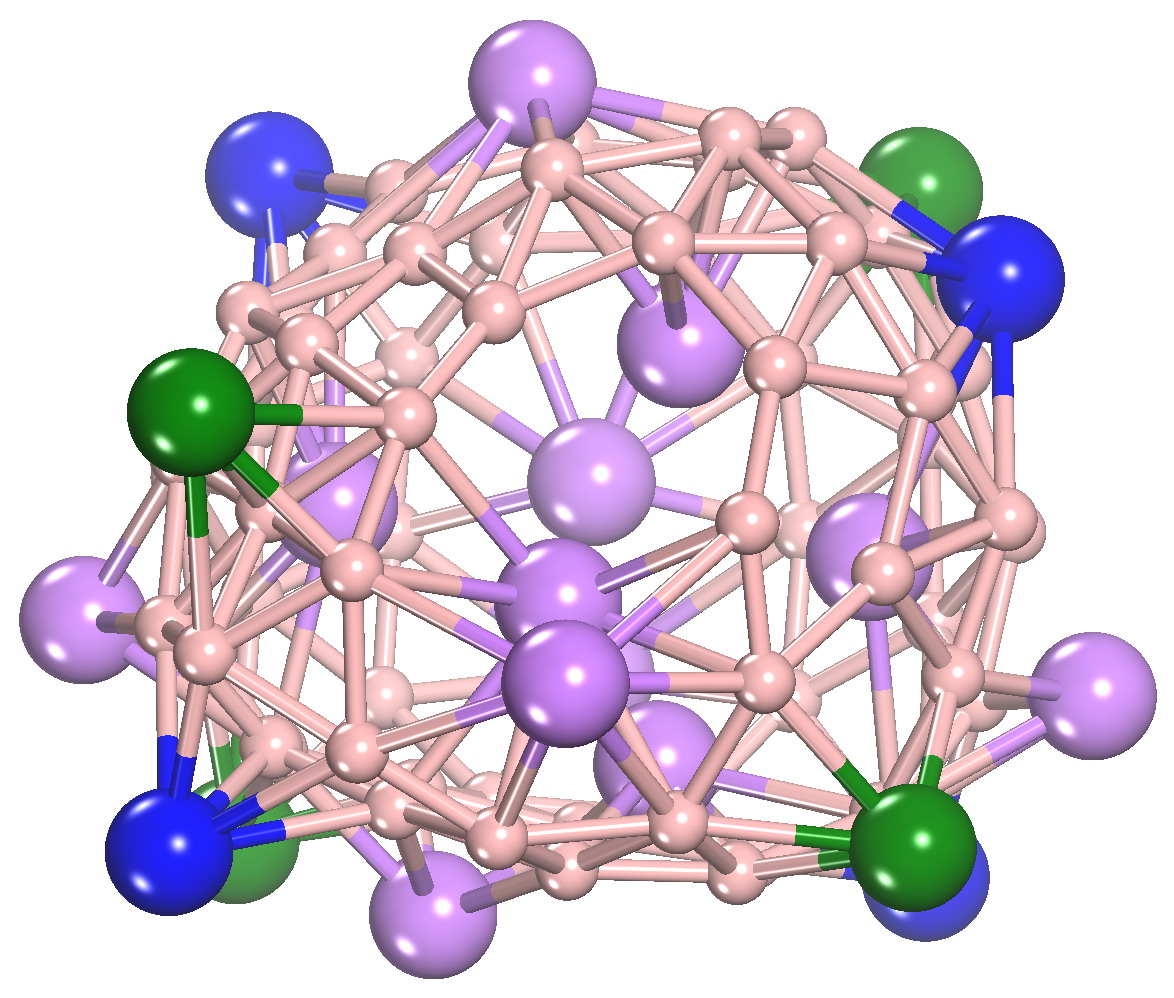 | 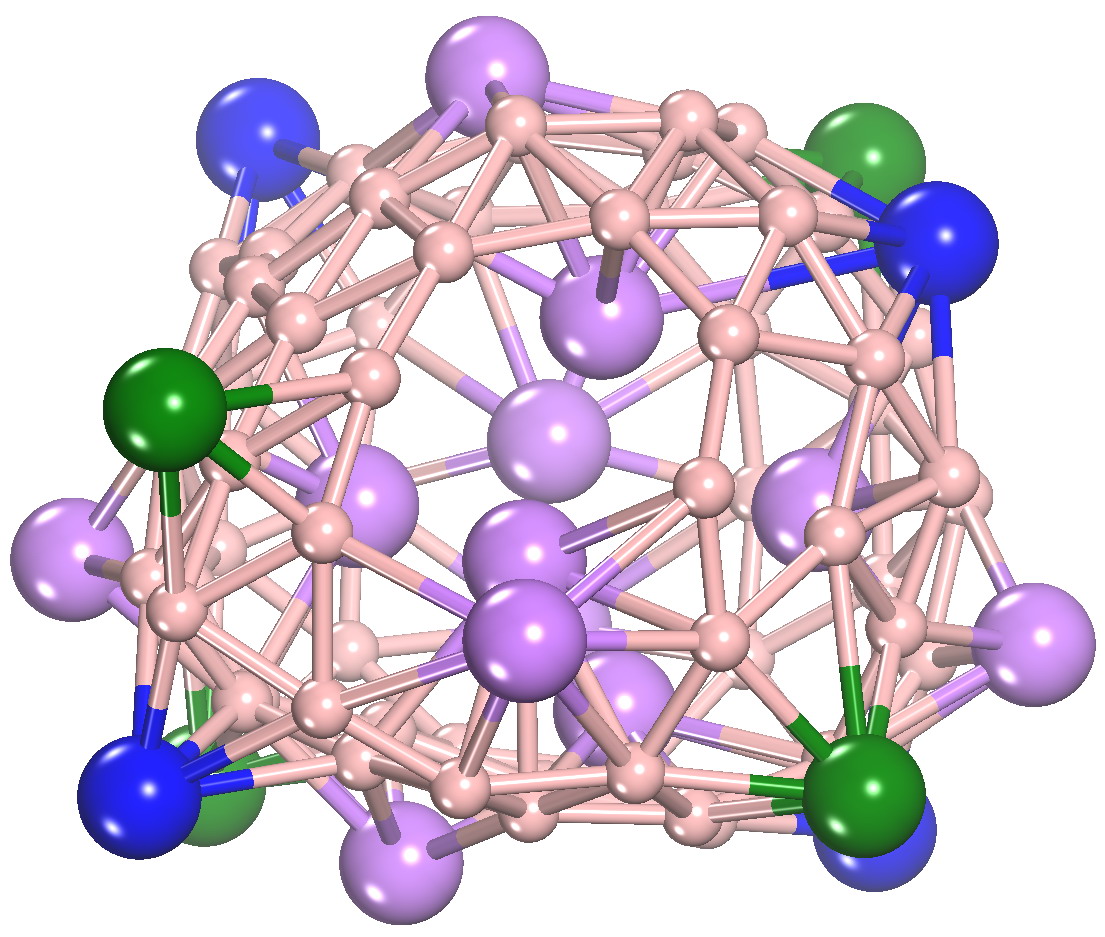 |
| (*e*) NVE-3000K-503*th*  ∆*E*= 2.963eV | (*f*) NVE-3000K-643*th*  ∆*E*= 2.164eV | (*g*) NVE-3000K-714*th*  ∆*E*= 1.759eV | (*h*) NVE-3000K-727*th*  ∆*E*= 1.503eV |
| 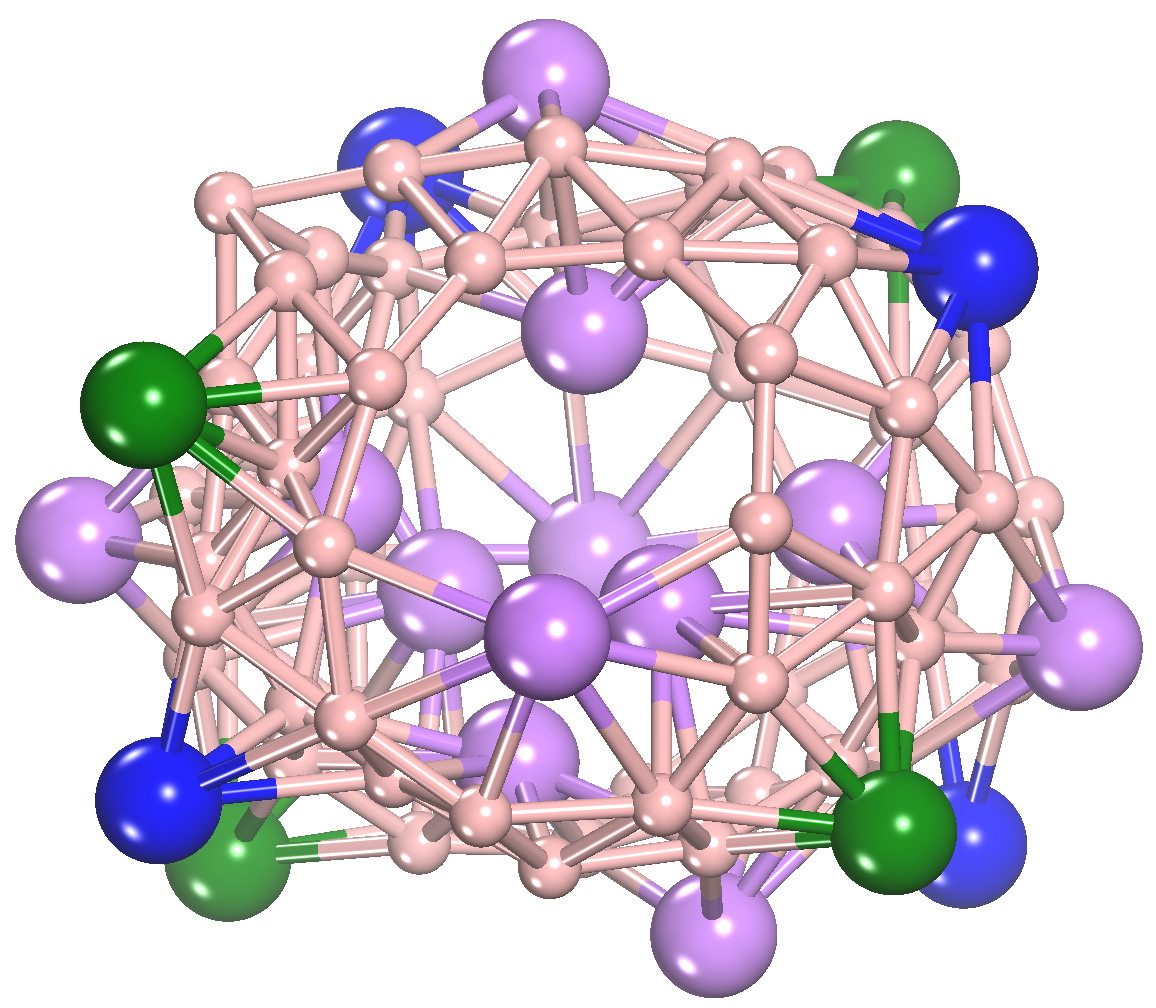 | 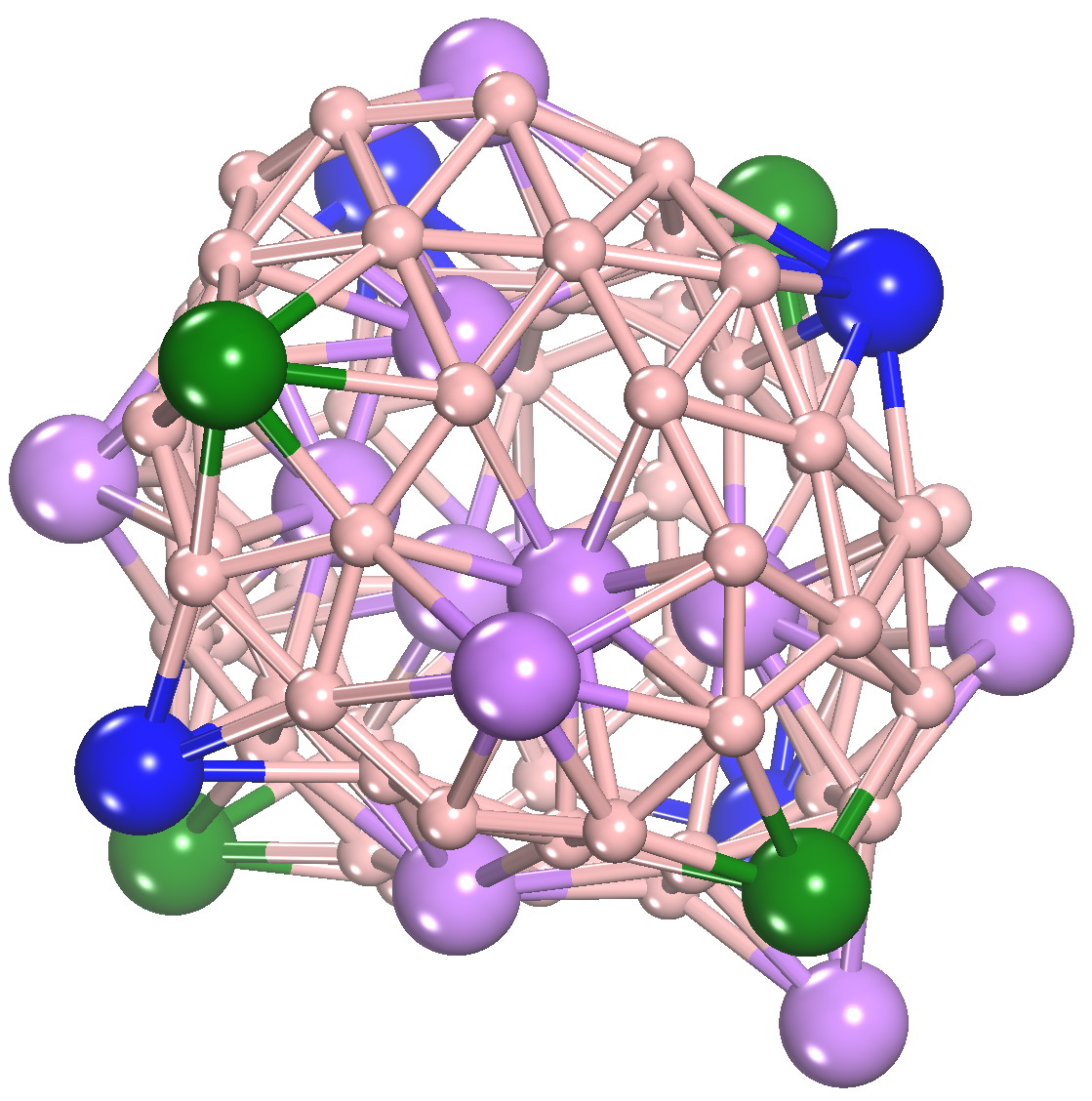 | 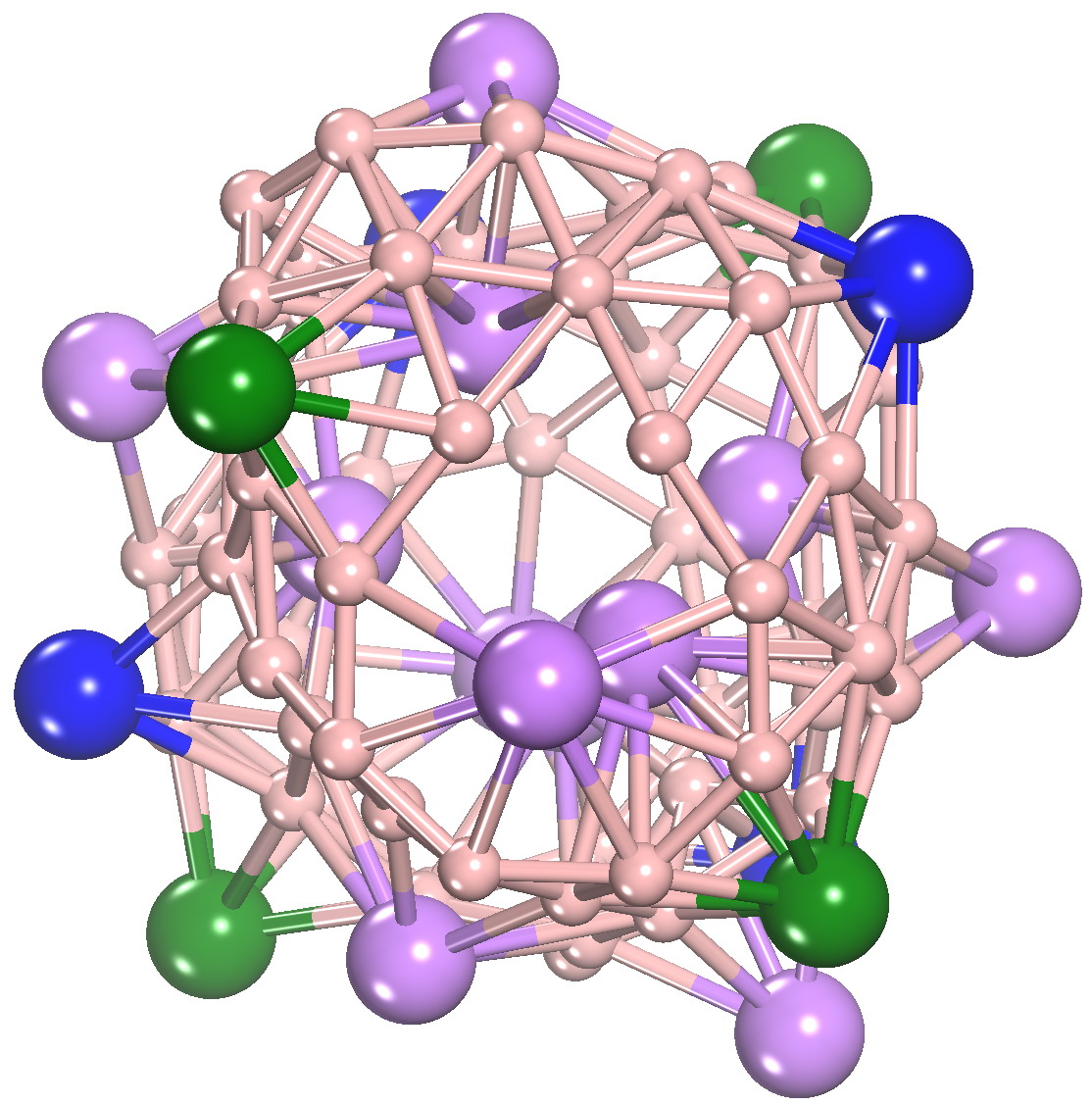 | 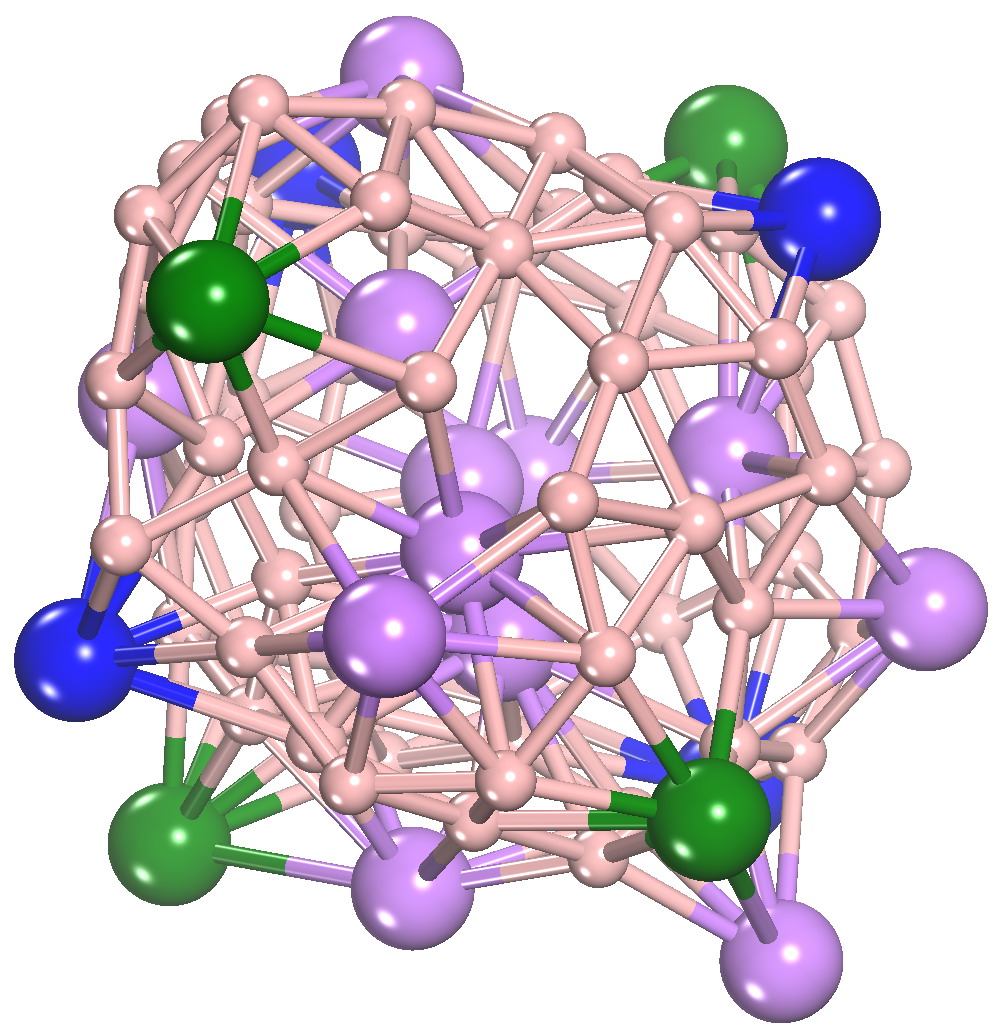 |
| (*i*) NVE-3000K-905*th*  ∆*E*= 1.986eV | (*j*) NVE-3000K-1055*th*  ∆*E*= 3.355eV | (*k*) NVE-3000K-1072*th*  ∆*E*= 3.775eV | (*l*) NVE-3000K-1329*th*  ∆*E*= 2.209eV |
| 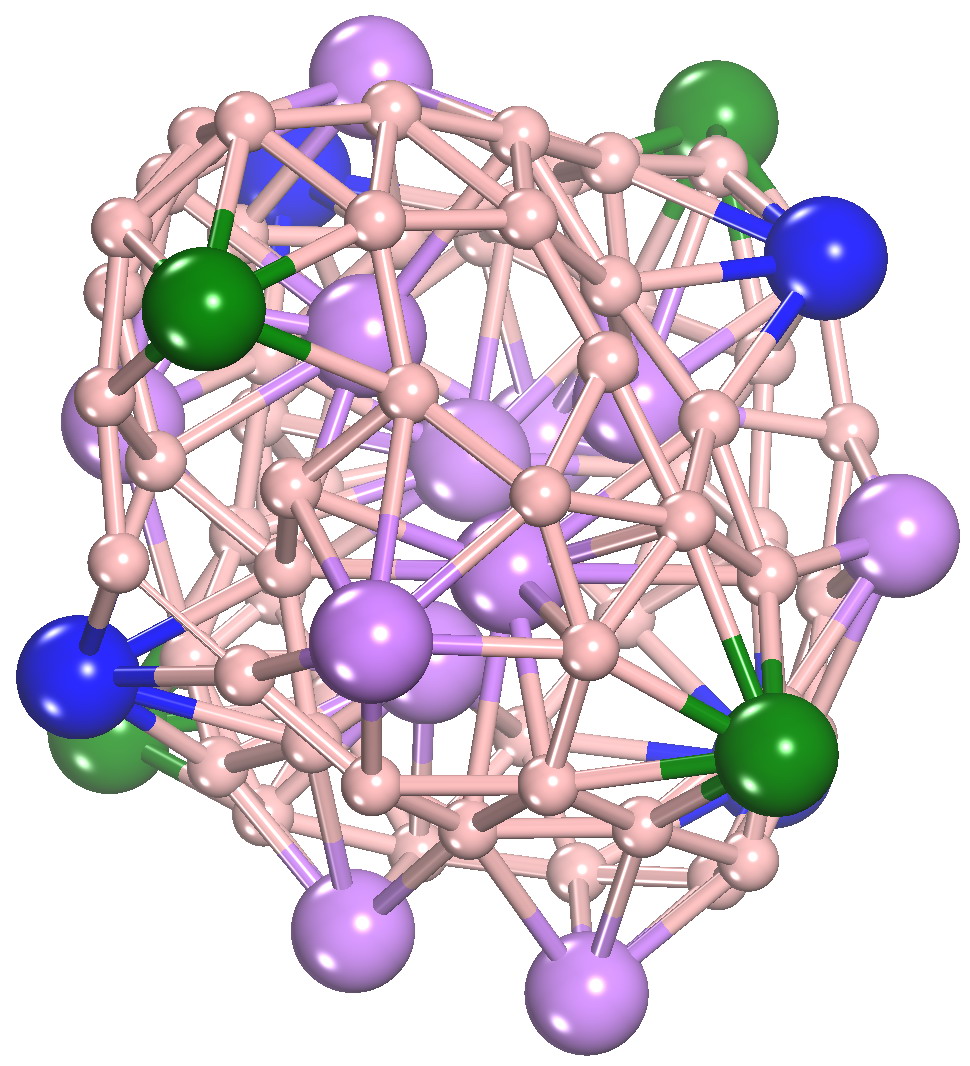 | 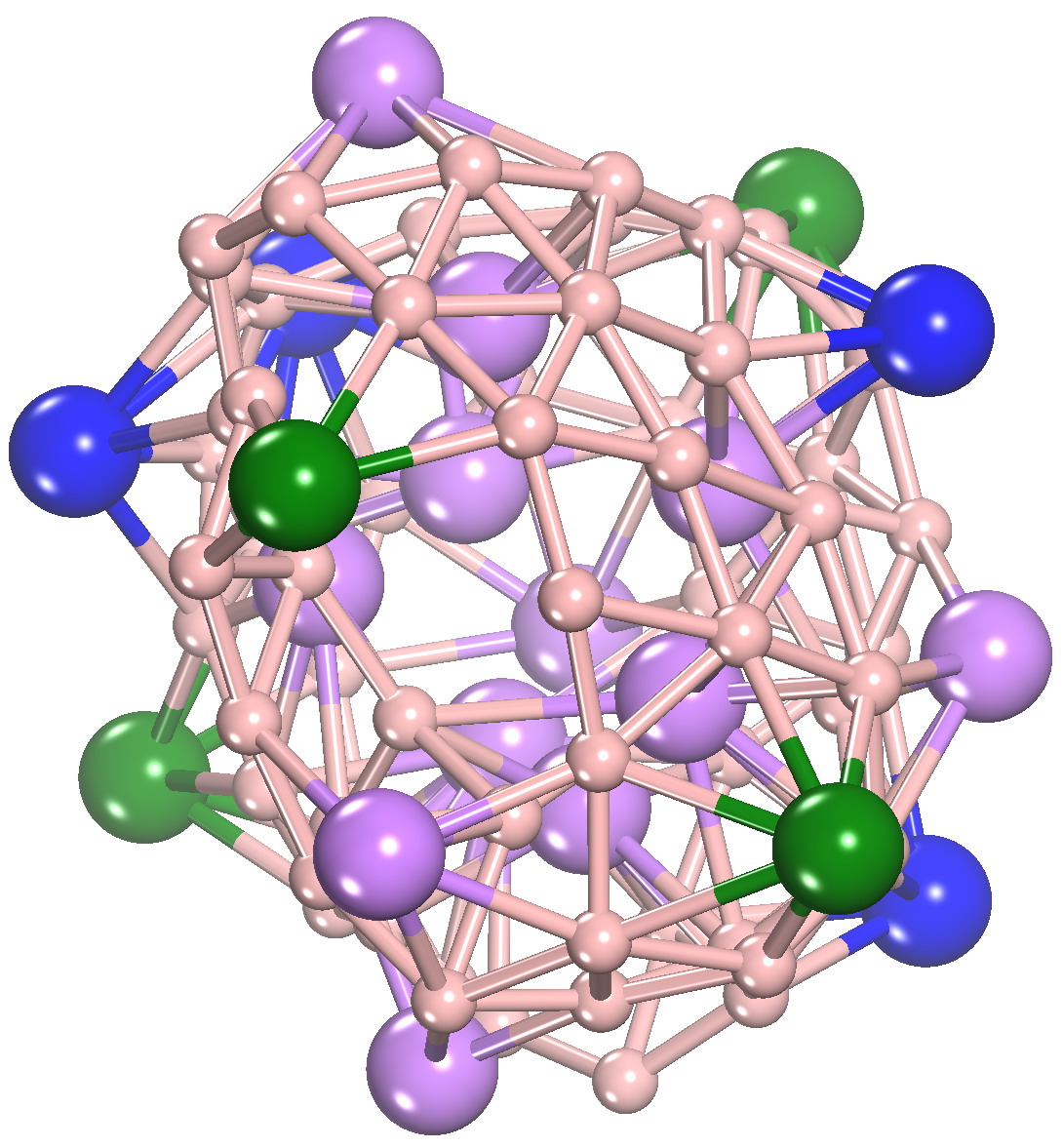 |  |  |
| (*m*) NVE-3000K-1604*th*  ∆*E*= 2.974eV | (*n*) NVE-3000K-2000*th*  ∆*E*= 2.009eV |  |  |
| **Figure S2.** The optimized configurations of Li_20_B_60_ clusters selected randomly from the NVE dynamic simulations with the temperature of 3000. Beneath each isomer is listed the relative energy (∆E) with respect to the D_2_-Li_20_B_60_. | | | |

|  |
| --- |

| | 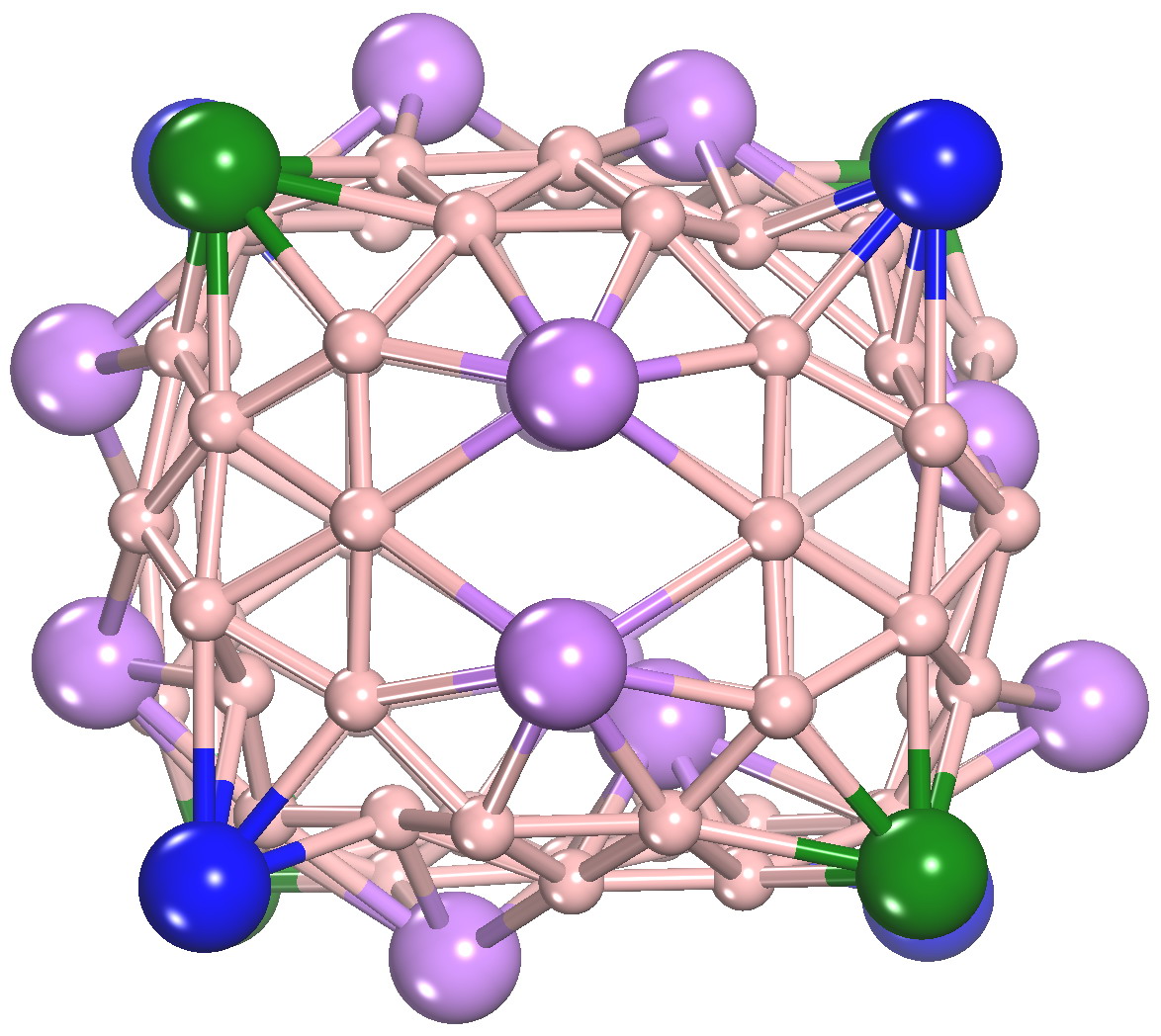 | 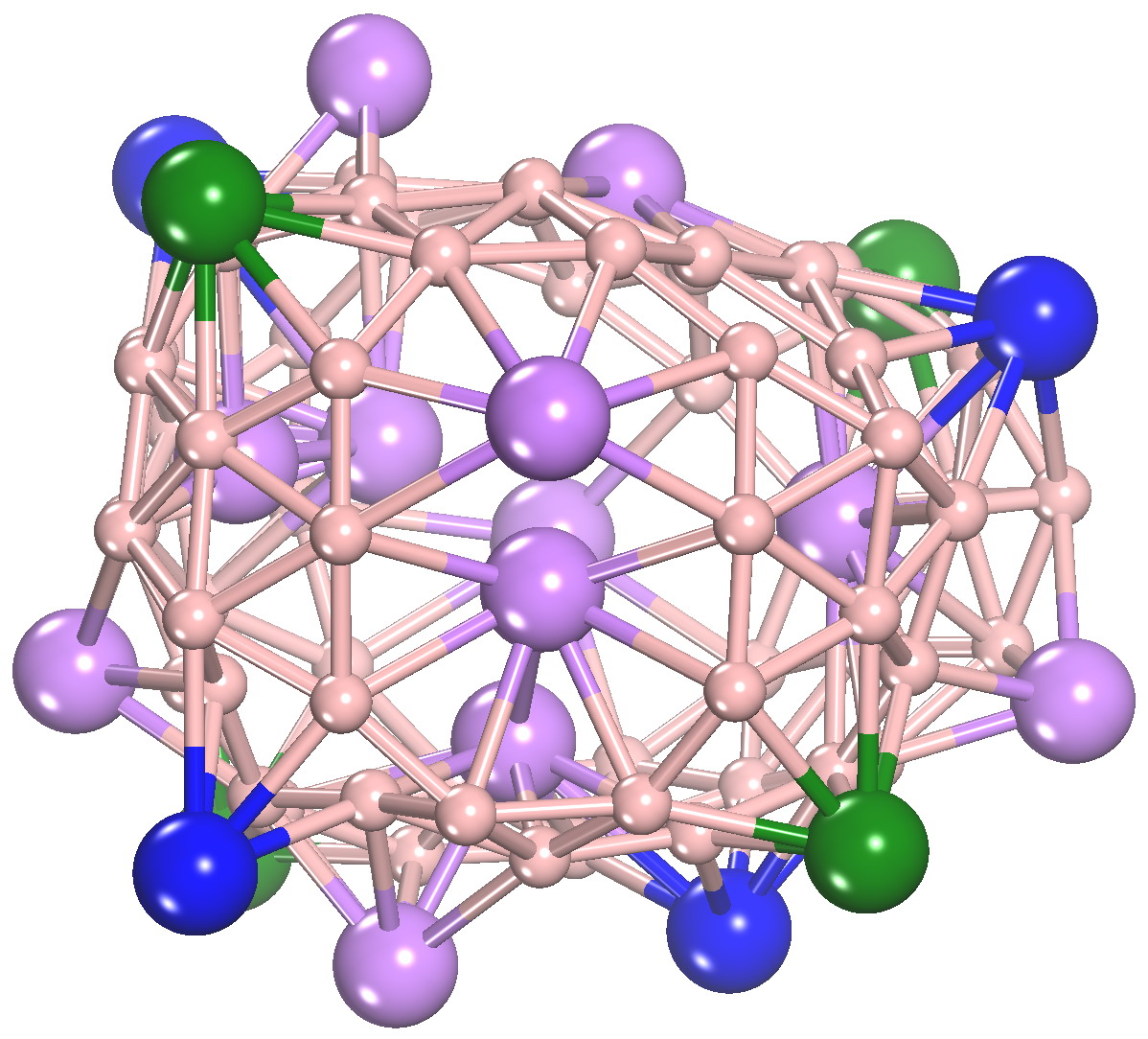 | 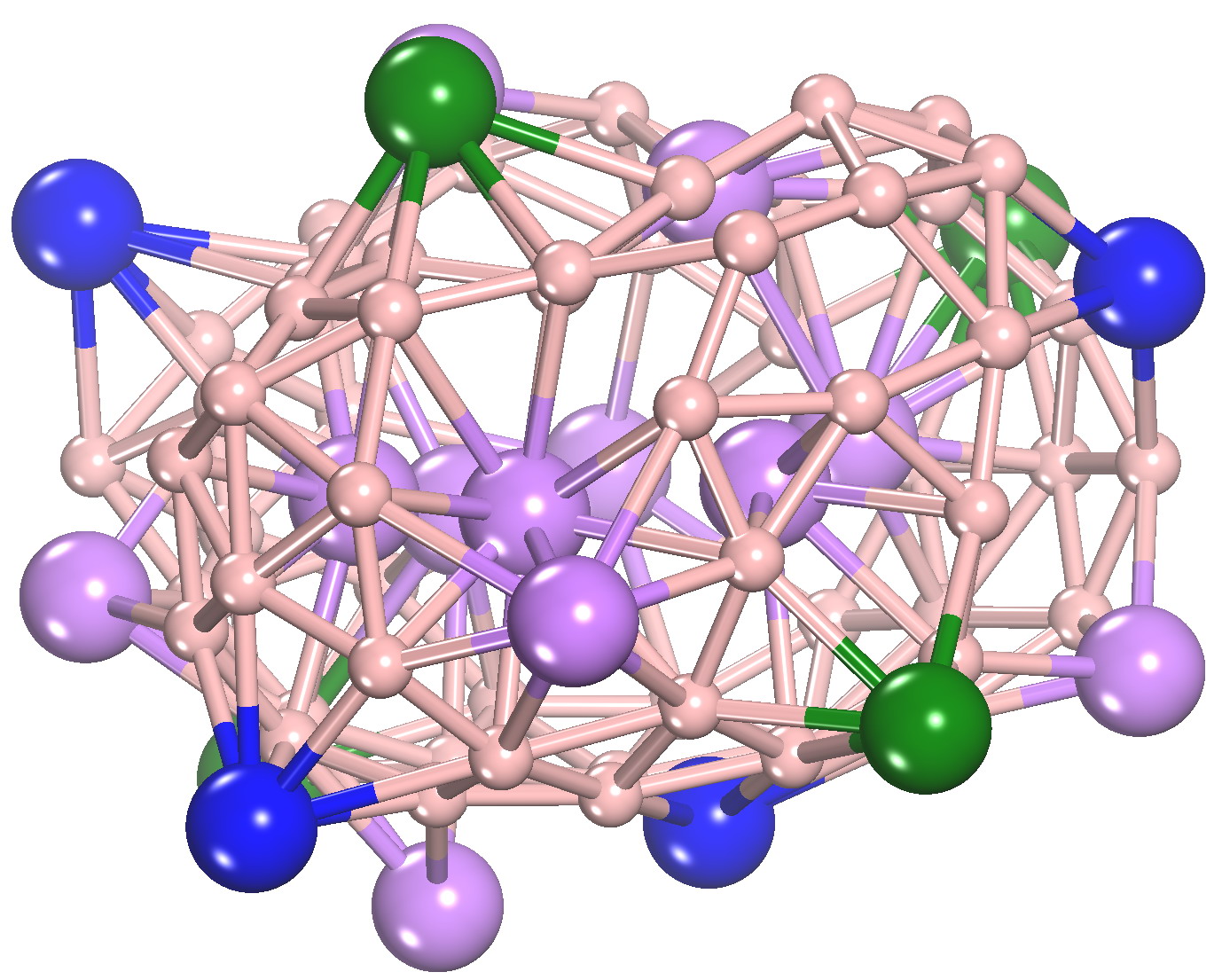 | 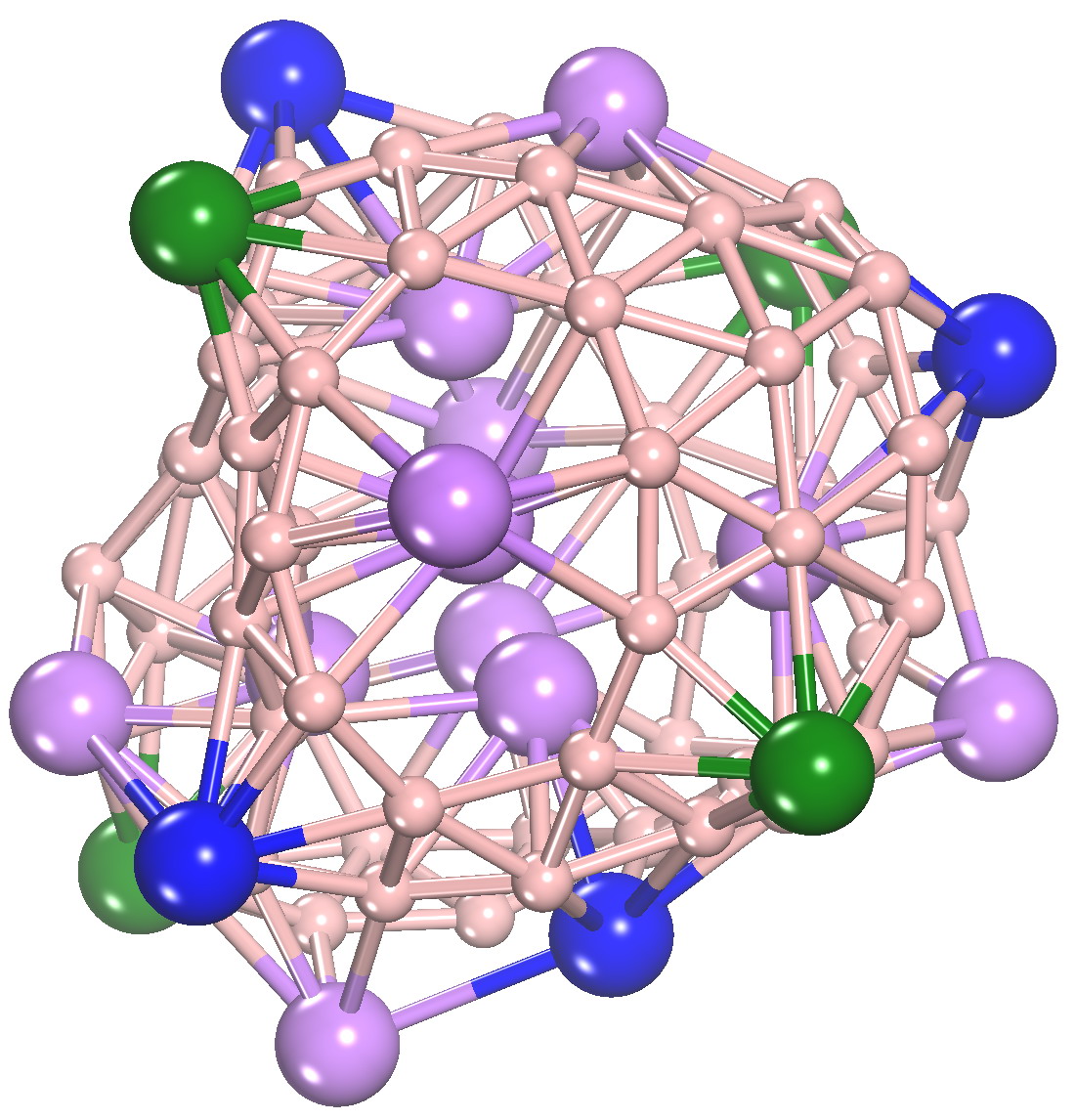 | | --- | --- | --- | --- | | (*a*) NVE-4000K-26*th*  ∆*E*= 0.555eV | (*b*) NVE-4000K-259*th*  ∆*E*= 2.971eV | (*c*) NVE-3000K-452*th*  ∆*E*= 2.151eV | (*d*) NVE-3000K-507*th*  ∆*E*= 4.537eV | | 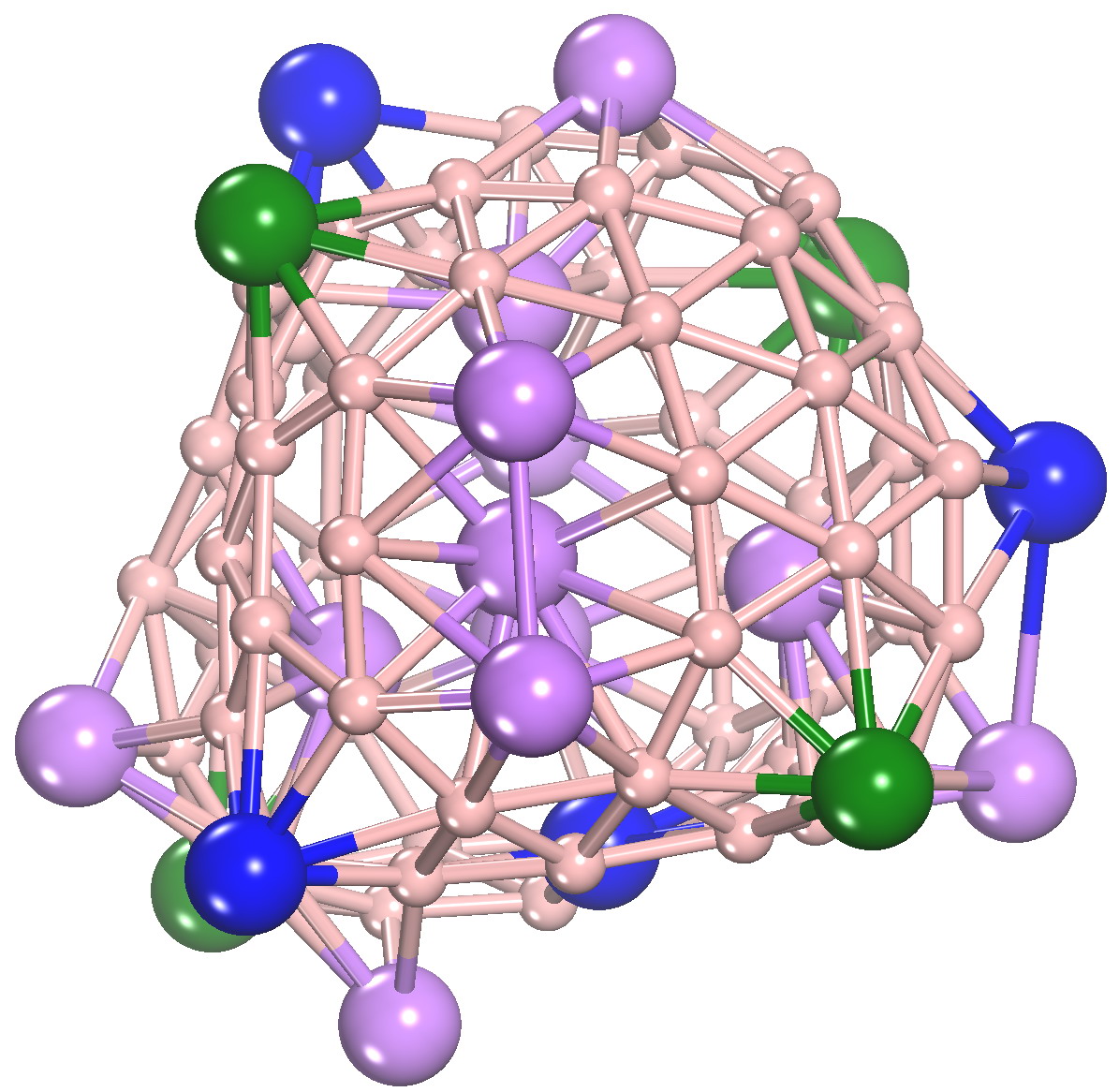 | 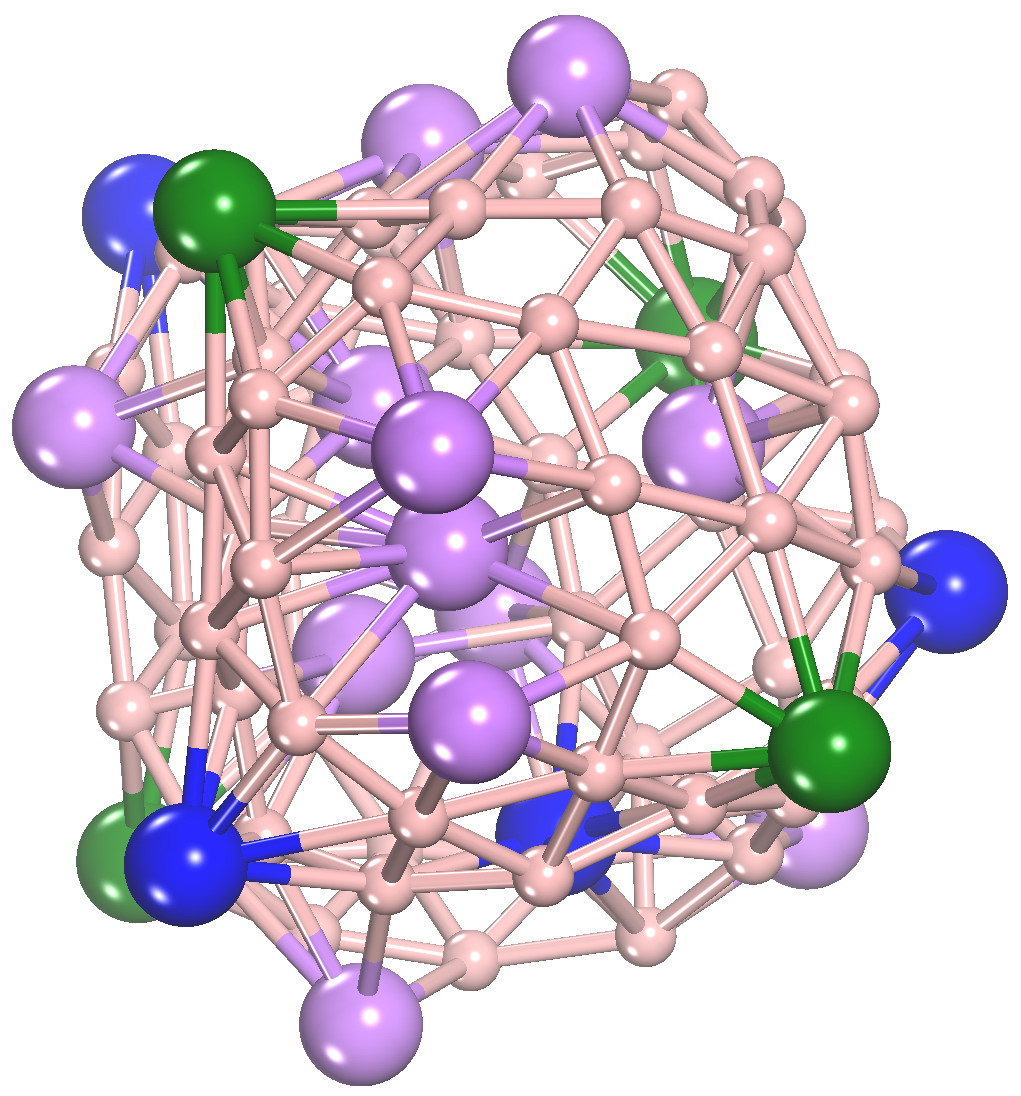 | 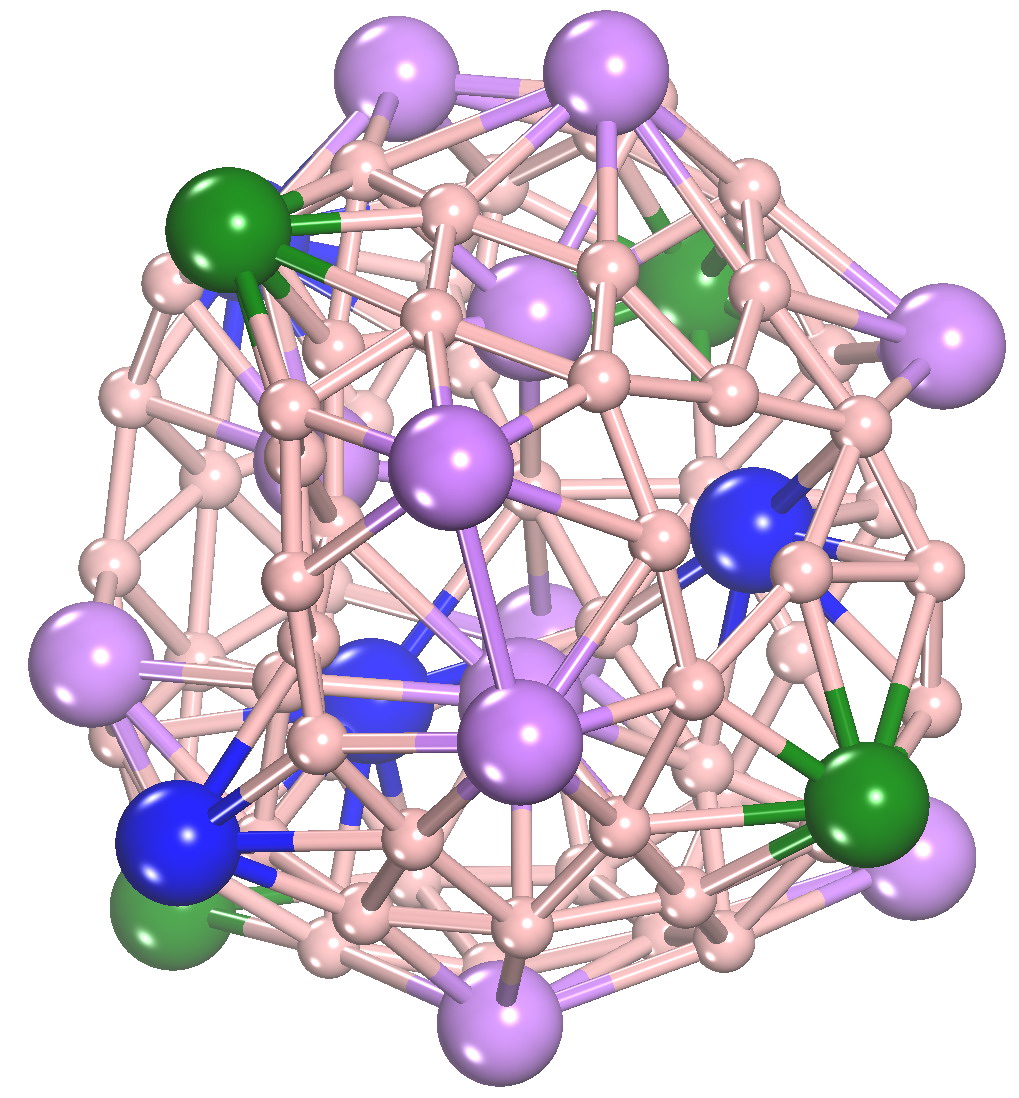 | 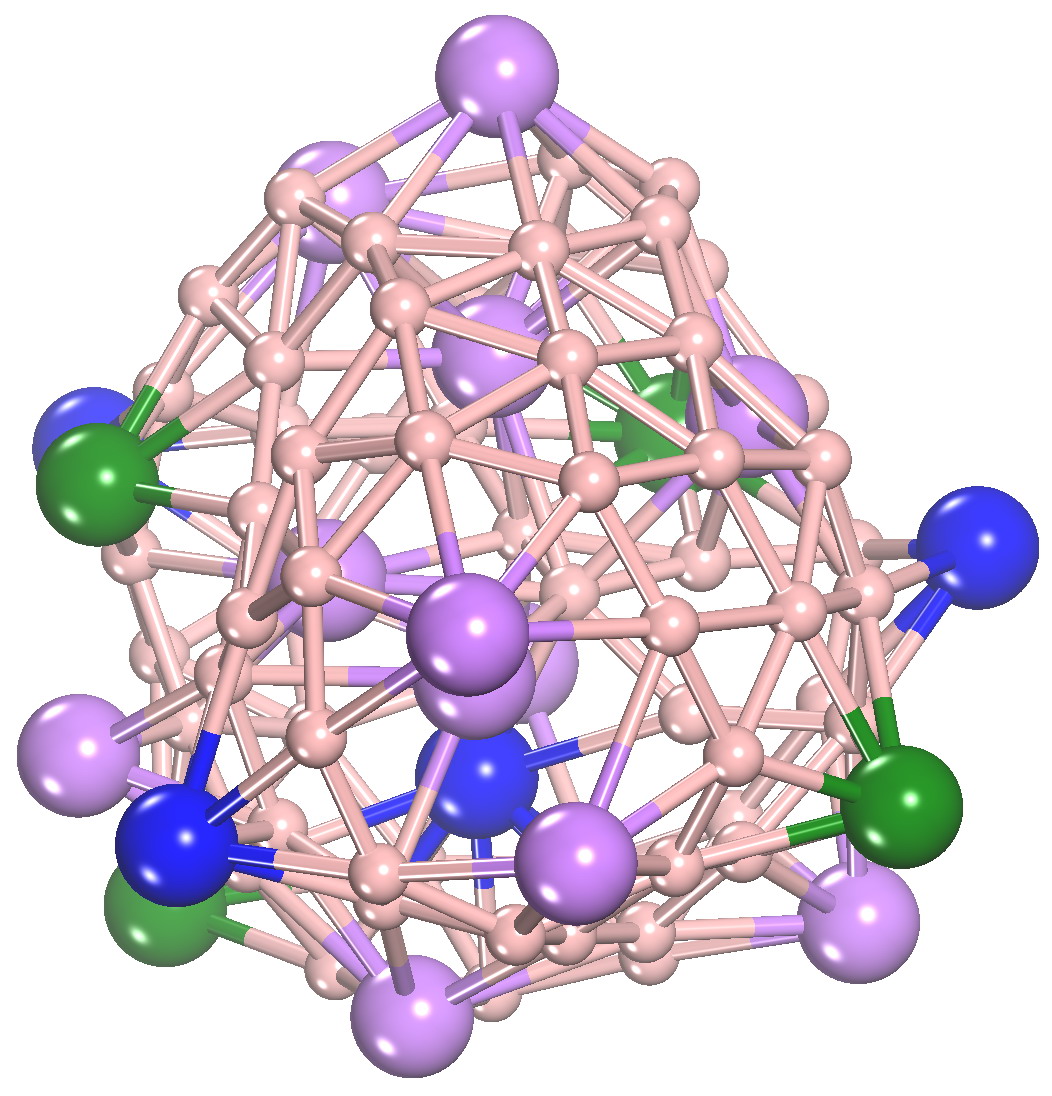 | | (*e*) NVE-4000K-619*th*  ∆*E*=4.480eV | (*f*) NVE-4000K-699*th*  ∆*E*= 2.430eV | (*g*) NVE-4000K-1025*th*  ∆*E*= 3.305eV | (*h*) NVE-4000K-1286*th*  ∆*E*= 4.288eV | | 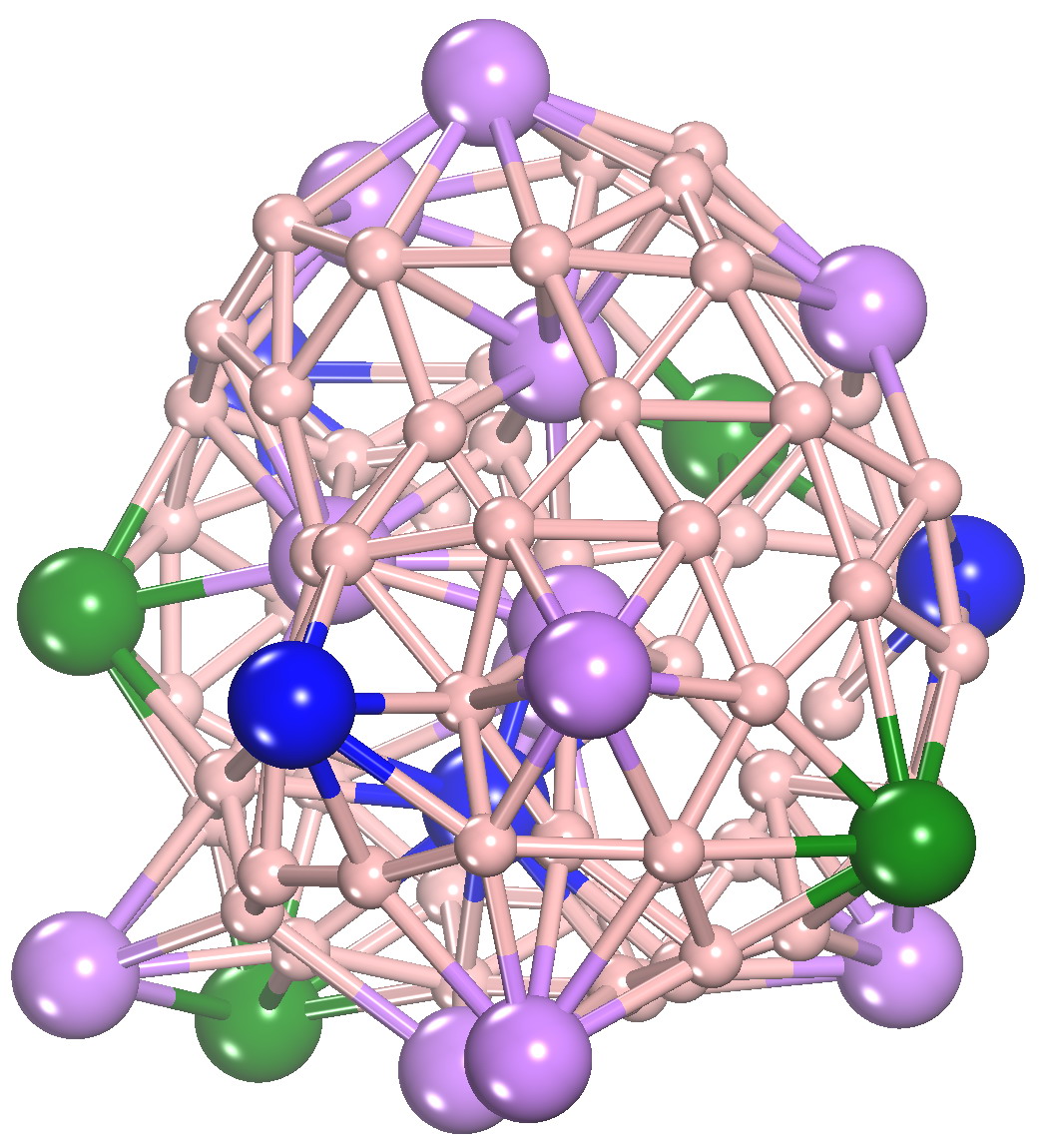 | 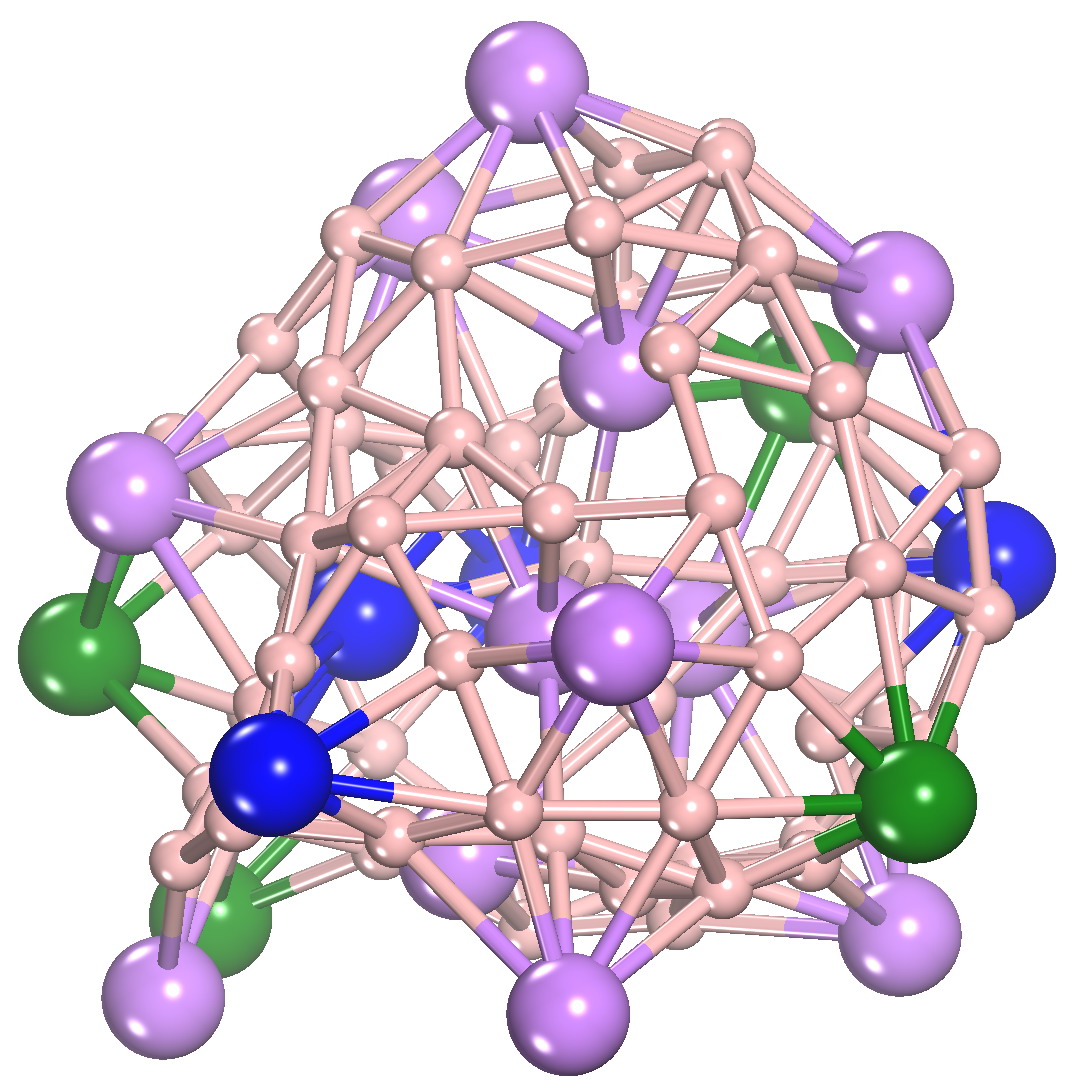 | 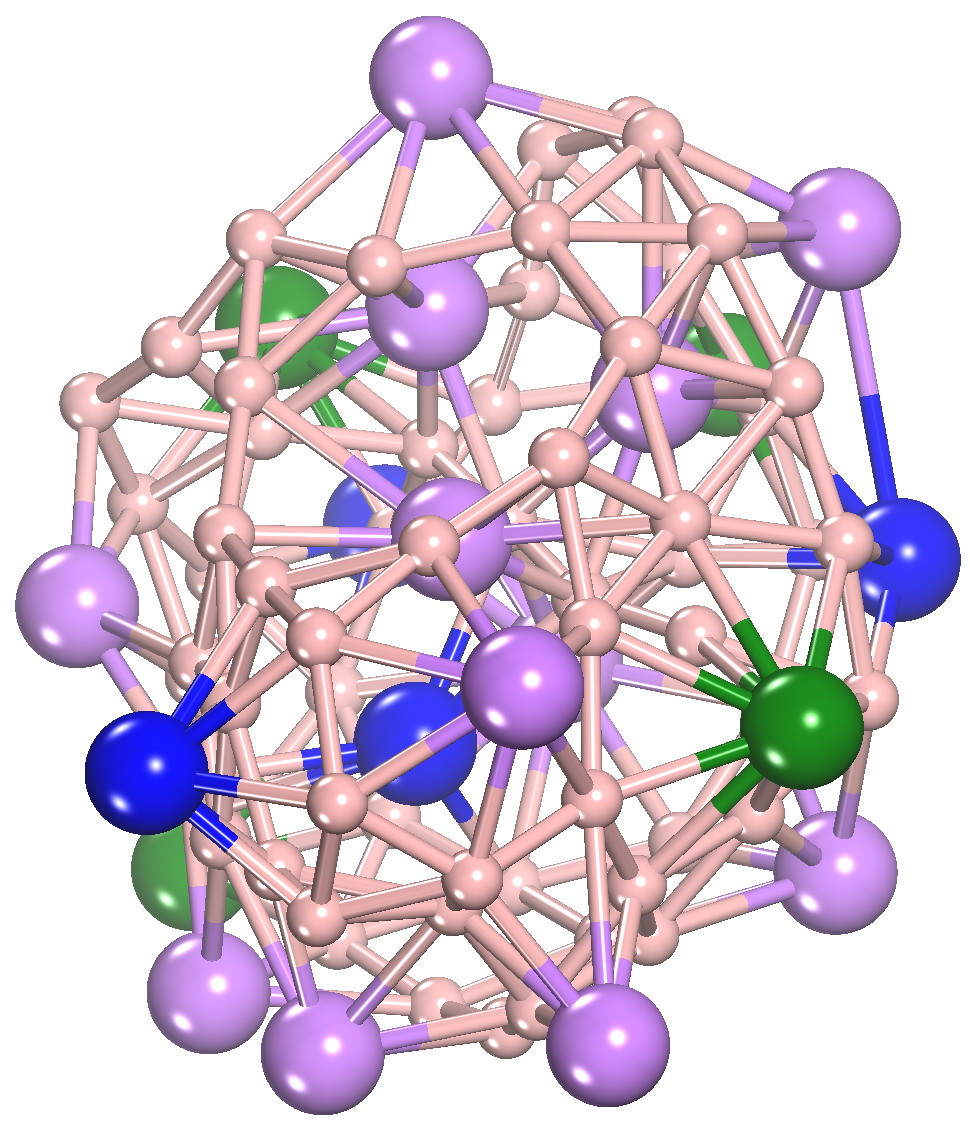 | 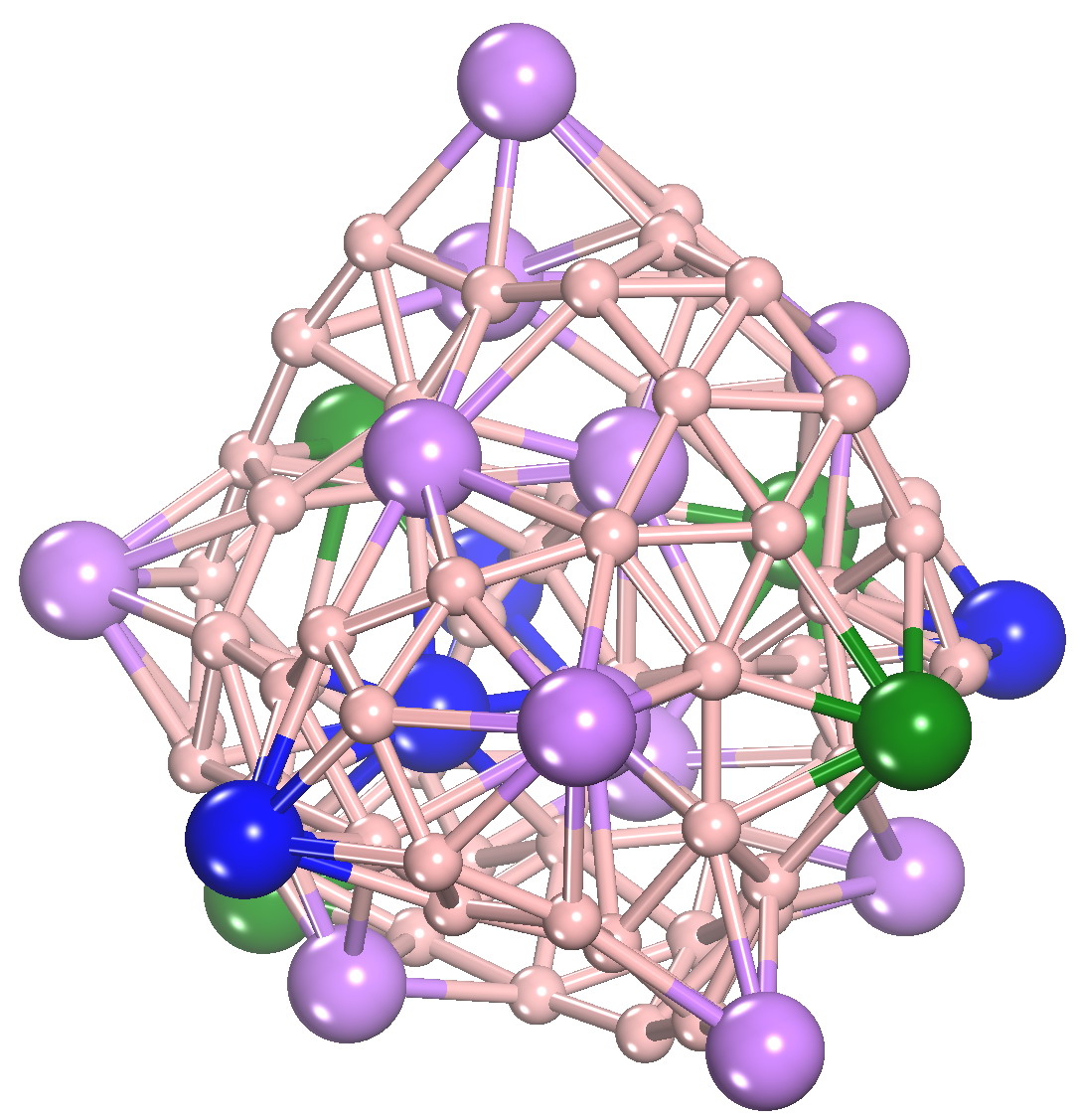 | | (*i*) NVE-4000K-1497*th*  ∆*E*= 1.635eV | (*j*) NVE-4000K-1542*th*  ∆*E*= 1.976eV | (*l*) NVE-3000K-1865*th*  ∆*E*= 1.856eV | (*n*) NVE-4000K-2000*th*  ∆*E*= 0.088eV | | **Figure S3.** The optimized configurations of Li_20_B_60_ clusters selected randomly from the NVE dynamic simulations with the temperature of 4000. Beneath each isomer is listed the relative energy (∆E) with respect to the D_2_-Li_20_B_60_. | | | | |
| --- | --- | --- | --- | --- | --- | --- | --- | --- | --- | --- | --- | --- | --- | --- | --- | --- | --- | --- | --- | --- | --- | --- | --- | --- | --- | --- | --- | --- |

**Section III. Hydrogen storage inside the Li_20_B_60_ cage**

As to the hydrogen storage inside the Li_20_B_60_ cage, the following two aspects were considered. 1) The process of a H_2_ molecule going into the Li_20_B_60_ cage was considered as shown in Figure S4. It is found that the energy barrier of a H_2_ molecule going into the Li_20_B_60_ cage through the gap between two Li^I^ atoms is about 1.88 eV. Although the energy barrier for actual situation is much lower when considering the temperature effect, it might be a little difficult for a H_2_ molecule going into Li_20_B_60_ in an interesting temperature range. 2) The H_2_ molecules were placed directly inside the Li_20_B_60_ cage. After optimization, it is found that these H_2_ molecules prefer the form of H atoms and the H atoms bond with the neighboring atoms as shown in Figure S5(*b*). It indicates that the inner of Li_20_B_60_ cage is unfavorable for reversible hydrogen storage.

|  |
| --- |
|  |
| **Figure S4.** Binding energy changes (eV) *vs.* distance *d* (Å) of the center of H_2_ molecule relative to the center of the opposite Li^I^-Li^I^ "face". The right axis gives the relative energy in eV. The plot is about the process of the atom going into Li_20_B_60_. |

| **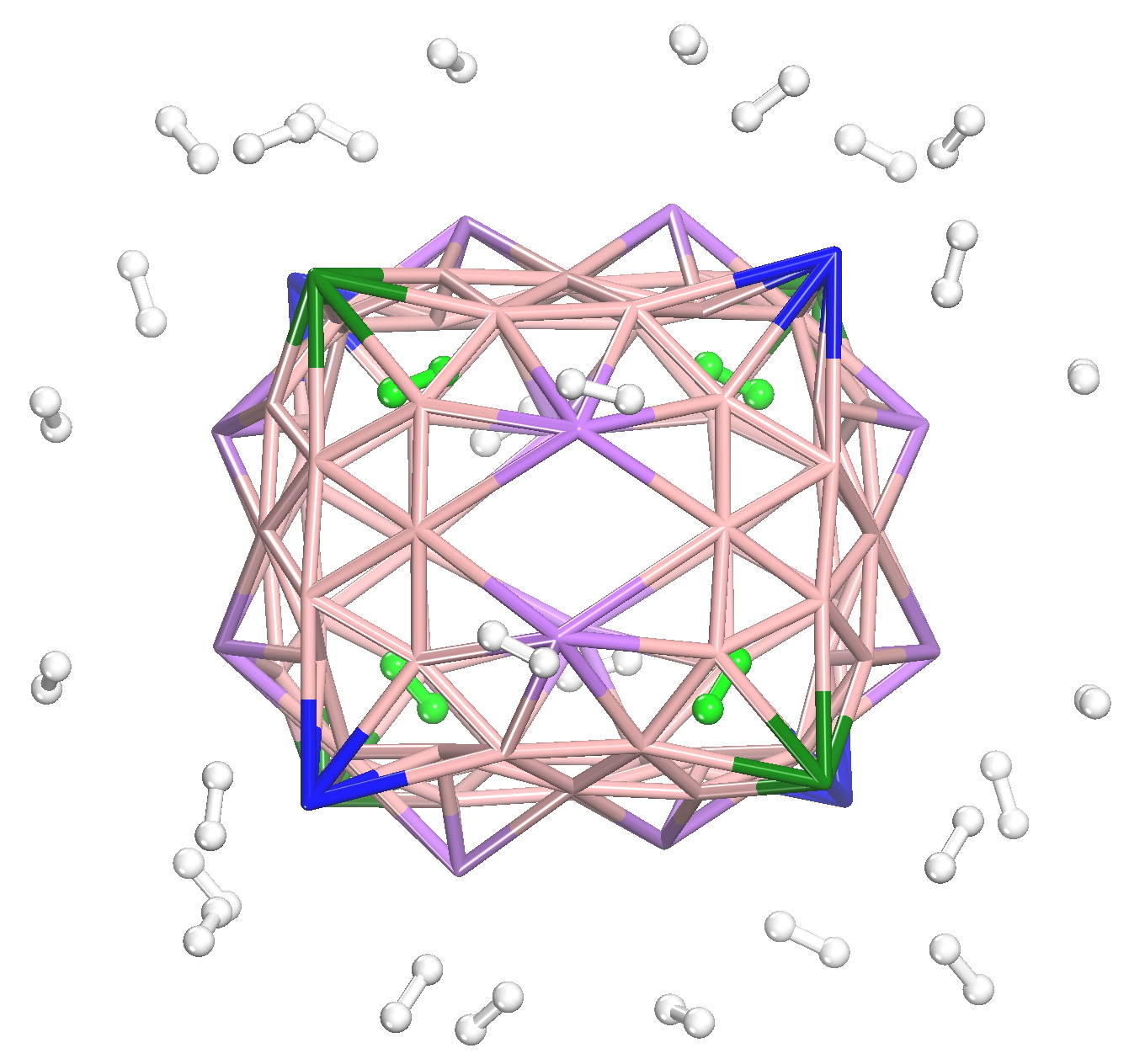** | **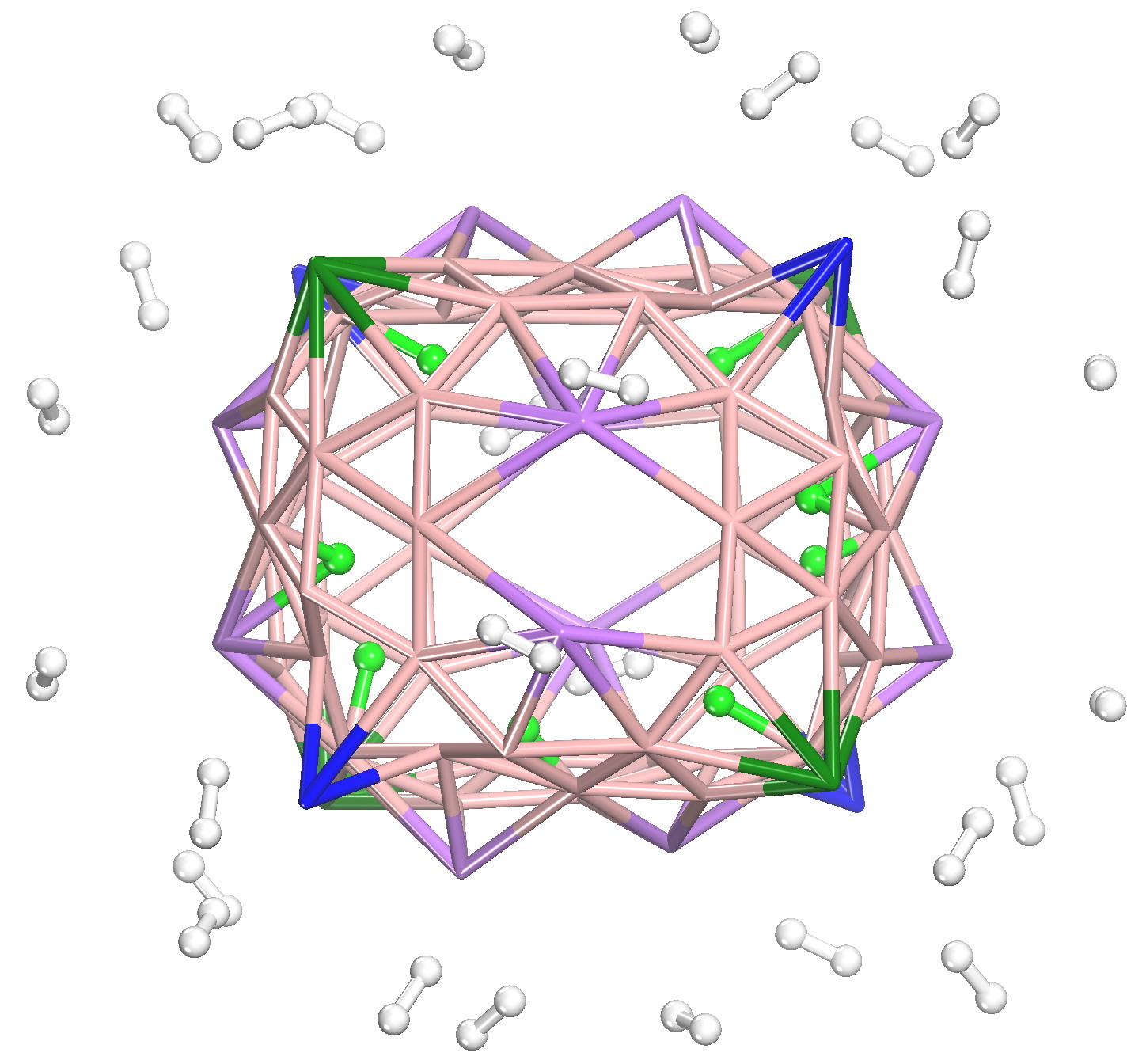** |
| --- | --- |
| (*a*) | (*b*) |
| **Figure S5.** Initial (*a*) and optimized (*b*) configurations of Li_20_B_60_ cage with hydrogen molecules inside (green) and outsider (white). | |

**Section IV. Vibrational frequency analysis and** **some low-frequency modes**

|  |
| --- |
| **Figure S6.** The vibrational frequency analysis for the Li_20_B_60_ cage. |
|  |
|  |
| **Figure S7.** The twelve low-frequency modes. Below each configuration list the values of frequency and the corresponding intensities. |
